# Inhibition of lysosome-tethered Ragulator-Rag-3D complex restricts the replication of Enterovirus 71 and Coxsackie A16

**DOI:** 10.1083/jcb.202303108

**Published:** 2023-10-31

**Authors:** Xinhui Wang, Zhilin Hu, Wei Zhang, Shuwei Wu, Yongjin Hao, Xia Xiao, Jingjing Li, Xiaoliang Yu, Chengkui Yang, Jingfeng Wang, Huiying Zhang, Feng Ma, Weifeng Shi, Jianwei Wang, Xiaobo Lei, Xiaohu Zhang, Sudan He

**Affiliations:** 1 State Key Laboratory of Common Mechanism Research for Major Diseases, and Key Laboratory of Synthetic Biology Regulatory Elements, Suzhou Institute of Systems Medicine, Chinese Academy of Medical Sciences and Peking Union Medical College, Suzhou, China; 2 Cyrus Tang Hematology Center and Collaborative Innovation Center of Hematology, Soochow University, Suzhou, China; 3 Jiangsu Key Laboratory of Neuropsychiatric Diseases and College of Pharmaceutical Sciences, Soochow University, Suzhou, China; 4 National Health Commission Key Laboratory of Systems Biology of Pathogens and Christophe Mérieux Laboratory, Institute of Pathogen Biology, Chinese Academy of Medical Sciences and Peking Union Medical College, Beijing, China; 5 Key Laboratory of Respiratory Disease Pathogenomics, Chinese Academy of Medical Sciences, Beijing, China; 6 National Key Laboratory of Immunity and Inflammation, and Key Laboratory of Synthetic Biology Regulatory Elements, Suzhou Institute of Systems Medicine, Chinese Academy of Medical Sciences and Peking Union Medical College, Suzhou, China; 7 Department of Laboratory Medicine, The Third Affiliated Hospital of Soochow University, Changzhou, China

## Abstract

Enterovirus 71 (EV71) and Coxsackie A16 (CVA16) are two major causative agents of hand, foot, and mouth disease (HFMD) in young children. However, the mechanisms regulating the replication and pathogenesis of EV71/CVA16 remain incompletely understood. We performed a genome-wide CRISPR-Cas9 knockout screen and identified Ragulator as a mediator of EV71-induced apoptosis and pyroptosis. The Ragulator-Rag complex is required for EV71 and CVA16 replication. Upon infection, the Ragulator-Rag complex recruits viral 3D protein to the lysosomal surface through the interaction between 3D and RagB. Disruption of the lysosome-tethered Ragulator-Rag-3D complex significantly impairs the replication of EV71/CVA16. We discovered a novel EV71 inhibitor, ZHSI-1, which interacts with 3D and significantly reduces the lysosomal tethering of 3D. ZHSI-1 treatment significantly represses replication of EV71/CVA16 as well as virus-induced pyroptosis associated with viral pathogenesis. Importantly, ZHSI-1 treatment effectively protects against EV71 infection in neonatal and young mice. Thus, our study indicates that targeting lysosome-tethered Ragulator-Rag-3D may be an effective therapeutic strategy for HFMD.

## Teaser

Wang et al. report that the Ragulator-Rag complex recruits EV71 3D protein to the lysosomal surface, facilitating the replication of EV71 and CVA16. ZHSI-1 was identified as a novel inhibitor of EV71 and CVA16 replication, which blocks lysosomal tethering of 3D. ZHSI-1 treatment effectively protects against EV71 infection in mice.

## Introduction

Hand, foot, and mouth disease (HFMD) is a common infectious disease among young children worldwide, especially in the Asia-Pacific region, where many severe outbreaks have occurred over the last decade (Chan et al., 2000, 2003; Schmidt et al., 1974; Solomon et al., 2010; Xing et al., 2014). HFMD is generally a self-limited illness, with symptoms including fever, mouth sores, and rashes on the hands and feet. However, severe cases can cause central nervous system diseases such as aseptic meningitis, encephalitis, acute flaccid paralysis, and even death (Chan et al., 2000; Chang et al., 1999; McMinn et al., 2001; McMinn, 2002; Tan and Chu, 2021). HMFD is caused by infection with enteroviruses, especially Enterovirus 71 (EV71) and Coxsackie A16 (CVA16; Chong et al., 2015). EV71 and CVA16 infection cause cytopathic effects associated with cell death. In EV71-infected cells, apoptosis is induced by the activation of the executioner caspase, capsase-3. Active caspase-3 cleaves gasdermin E (GSDME), thus switching from caspase-3-mediated apoptosis to GSDME-mediated pyroptosis (Dong et al., 2022). As an inflammatory form of cell death, GSDME-mediated pyroptosis contributes to the pathogenesis of EV71 infection involving the central nervous system (Dong et al., 2022). Owing to the lack of effective anti-enterovirus drugs for treating HFMD, there remains a significant unmet medical need to develop antiviral strategies against these infections.

Enteroviruses are small, single-stranded, positive-sense RNA viruses that belong to the *Picornaviridae* family (Schmidt et al., 1974). EV71 and CVA16 enter cells by endocytosis through specific receptors, including human P-selectin glycoprotein ligand-1 (PSGL-1), scavenger receptor B2 (SCARB2), and glycan receptors (Baggen et al., 2019; Nishimura et al., 2009; Yamayoshi et al., 2009). The viral RNA of EV71 and CVA16 is directly translated into a large polyprotein, which is cleaved into capsid and non-structural proteins by viral proteases (Pöyry et al., 1994; Solomon et al., 2010). Among these, the RNA-dependent RNA polymerase (RdRP), also known as 3D, is essential for the replication of the viral RNA genome. EV71 3A and 3D interact with host factors, including phosphatidylinositol 4-kinase III (PI4KB), to form phosphatidylinositol 4-phosphate (PI4P)-enriched replication organelles (ROs) that facilitate viral replication (Baggen et al., 2018; Hsu et al., 2010; Lyoo et al., 2019; Xiao et al., 2017). Also, increasing evidence suggests that enterovirus proteins hijack host factors involved in membrane trafficking and biosynthesis pathways to promote efficient viral genome replication (Hsu et al., 2010; Kim and Bergelson, 2012; McPhail et al., 2020; Morosky et al., 2016). However, how these host factors are hijacked by enteroviral proteins to support EV71 and CVA16 replication is incompletely understood.

Ragulator is a heteropentameric protein complex composed of two heterodimers, LAMTOR2/LAMTOR3 and LAMTOR4/LAMTOR5, and a scaffold protein, LAMTOR1 (Mu et al., 2017). LAMTOR1 wraps LAMTOR2 to LAMTOR5 and anchors the Ragulator complex to the lysosomal membrane (Mu et al., 2017; Nada et al., 2009). Ragulator then tethers the Rag GTPase heterodimers, RagA/B and RagC/D, to the lysosomal membrane and acts as a guanylate exchange factor by activating Rag GTPase, which is essential for amino acid–mediated activation of mTORC1 (Bar-Peled et al., 2012; Sancak et al., 2008, 2010; Su et al., 2017). Activation of mTORC1 in response to cellular nutrients by lysosome-tethered Ragulator-Rag complex promotes biosynthetic processes, including protein and lipid synthesis and cell growth (Ben-Sahra and Manning, 2017; Dibble and Manning, 2013; Hosios et al., 2022). In addition, the Ragulator-Rag complex plays a crucial role in lysosomal trafficking and cell motility (Nakatani et al., 2021; Schiefermeier et al., 2014; Takahashi et al., 2012). Despite the importance of Ragulator-Rag in metabolic signaling and lysosomal trafficking, it remains unknown whether the Ragulator-Rag complex is involved in the replication of enteroviruses.

In this study, we performed a genome-wide genetic screen to identify host factors that participate in EV71 infection. We identified Ragulator as an essential factor for EV71-associated cell death. Subsequent analyses revealed that the lysosome-tethered Ragulator-Rag complex recruits viral 3D protein and PI4KB, and that this facilitates EV71/CVA16 replication. Finally, we show that inhibition of 3D lysosomal tethering with the novel compound, ZHSI-1, efficiently inhibits EV71 and CVA16 replication and viral pathogenesis in vitro and robustly attenuated clinical symptoms of EV71 infection in neonatal and young mice.

## Results

### Loss of Ragulator components blocks EV71-induced apoptosis and pyroptosis

**Figure S1.**
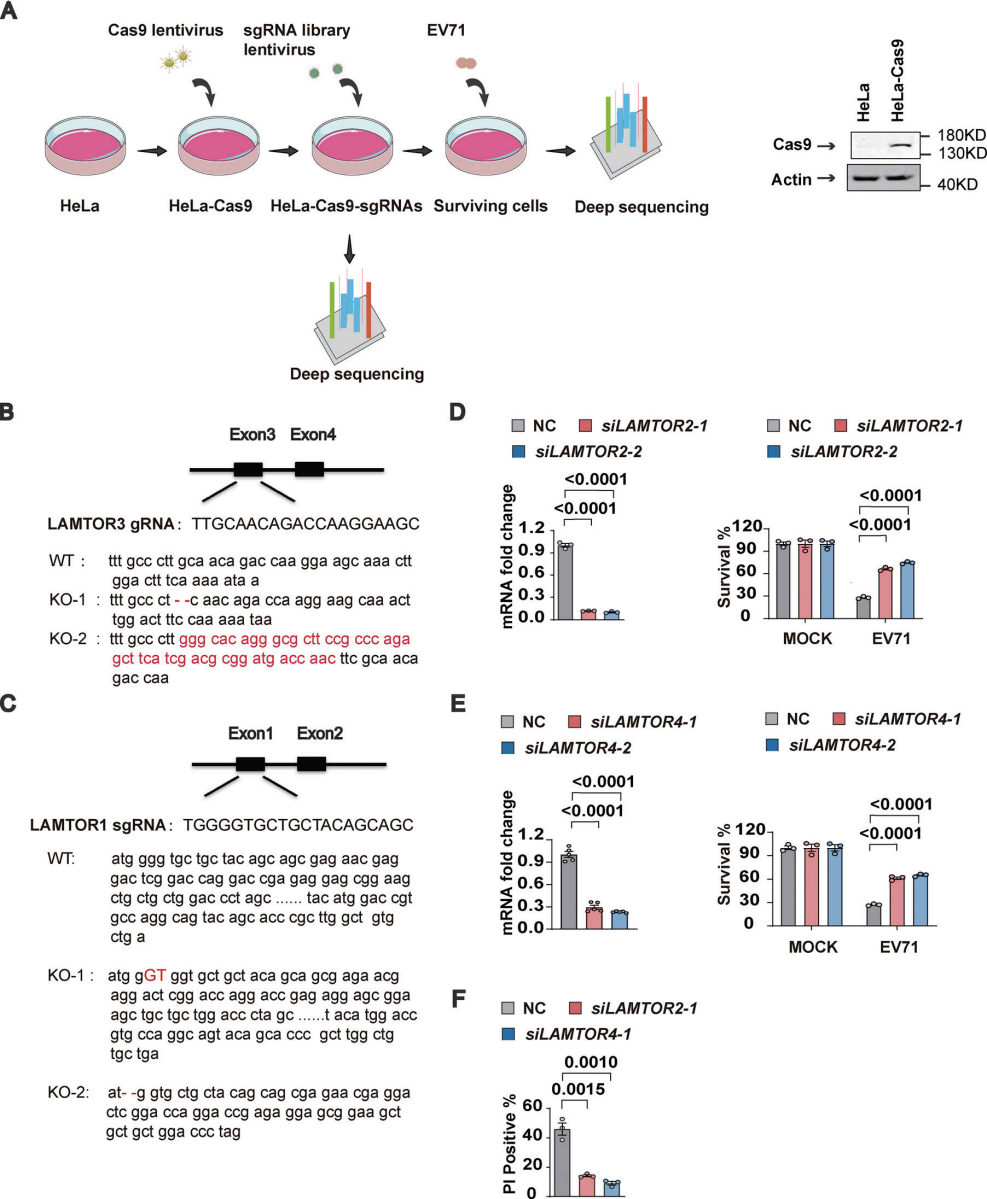
**Loss of Ragulator components blocks EV71-induced apoptosis and pyroptosis. (A)** Schematic diagram of the genome-wide CRISPR screening process and Western blot analysis of Cas9 expression in HeLa cells. **(B and C)** Sequencing analysis of knockout efficiency of *LAMTOR3* and *LAMTOR1*. **(D–F)** *LAMTOR2* and *LAMTOR4* were silenced in HeLa cells using siRNAs and then the indicated HeLa cells were infected with EV71 (MOI = 5) 60 h after transfection. The knockdown efficiency of *LAMTOR2* (D) and *LAMTOR4* (E) was determined by qPCR 60 h after transfection. Cell death induced by EV71 was detected by measuring ATP levels (D and E) and PI staining at 18 h after infection (F). All values are means ± SEM. Data were analyzed using two-tailed Student’s *t* test. Source data are available for this figure: SourceData FS1.

**Figure 1.**
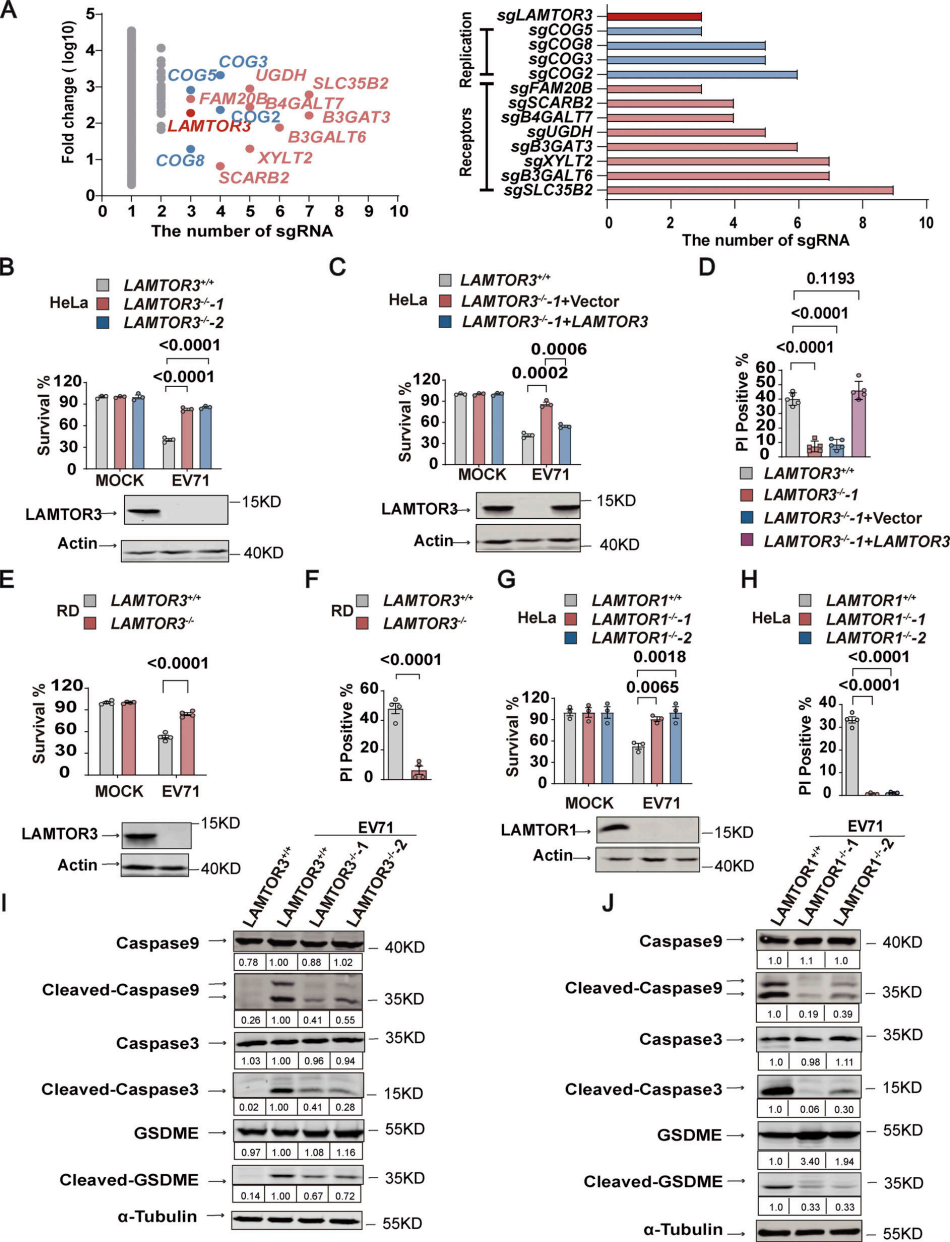
**Loss of Ragulator components blocks EV71-induced apoptosis and pyroptosis. (A)** A genome-wide CRISPR screening identified a total of 13 genes in which more than three sgRNAs were expressed at least fivefold higher in EV71-infected cells compared to mock-infected cells (left panel). In addition to *LAMTOR3*, other hits were identified that were either involved in enterovirus replication or acted as enterovirus receptors (right panel). The light red color represents genes that serve as receptors for enteroviruses. The blue color represents genes associated with enterovirus replication. The deep red color represents *LAMTOR3*. **(B)** *LAMTOR3*^*+/+*^ or *LAMTOR3*^*−/−*^ HeLa cells were infected (MOI = 5) with EV71 for 18 h and cell death was determined by measuring ATP levels (upper panel). The KO efficiency of *LAMTOR3* in HeLa cells was assessed by Western blot analysis (lower panel). All values are means ± SEM. Data were analyzed using a two-tailed Student’s *t* test. **(C)** *LAMTOR3*^*+/+*^ or *LAMTOR3*^*−/−*^ HeLa cells stably transfected with an empty vector (*LAMTOR3*^*−/−*^-1+Vector) or a vector expressing *LAMTOR3* (*LAMTOR3*^*−/−*^-1**+***LAMTOR3*) were infected with EV71 (MOI = 5) for 18 h and cell death was determined by measuring ATP levels (upper panel). The rescue efficiency of ectopic expression of *LAMTOR3* in *LAMTOR3*-KO HeLa cells was analyzed by Western blotting (lower panel). All values are means ± SEM. Data were analyzed using two-tailed Student’s *t* test. **(D)** HeLa cells as indicated (*LAMTOR3*^*+/+*^, *LAMTOR3*^*−/−*^-1, *LAMTOR3*^*−/−*^-1+Vector, and *LAMTOR3*^*−/−*^-1+*LAMTOR3*) were infected with EV71 (MOI = 5) for 18 h and cell death was determined by PI staining. All values are means ± SEM. Data were analyzed using two-tailed Student’s *t* test. **(E and F)** *LAMTOR3*^*+/+*^ or *LAMTOR3*^*−/−*^ RD cells were infected with EV71 (MOI = 2) for 18 h and cell death was detected by measuring ATP levels (E) and PI staining (F). The knockout efficiency of *LAMTOR3* in RD cells was determined by Western blot analysis (E). All values are means ± SEM. Data were analyzed using two-tailed Student’s *t* test. **(G and H)** *LAMTOR1*^*+/+*^ or *LAMTOR1*^*−/−*^ HeLa cells were infected with EV71 (MOI = 5) for 18 h and cell death was detected by measuring ATP levels (G) and PI staining (H). The knockout efficiency of *LAMTOR1* in HeLa cells was determined by Western blot analysis (G). All values are means ± SEM. Data were analyzed using two-tailed Student’s *t* test. **(I and J)** *LAMTOR3*^*+/+*^ and *LAMTOR3*^*−/−*^ (I) or *LAMTOR1*^*+/+*^ and *LAMTOR1*^*−/−*^ (J) HeLa cells were infected with EV71 (MOI = 5). Cleavage of GSDME and the activation of caspase3 and caspase9 were analyzed by Western blot 12 h after infection. The quantitation analysis of Western blot was performed using ImageJ. Source data are available for this figure: SourceData F1.

To identify host factors involved in EV71 replication and pathogenicity, we performed a genome-wide CRISPR-Cas9 knockout screen. HeLa cells stably expressing Cas9 were transduced with lentivirus expressing a pooled population of gRNAs targeting 18,543 genes, followed by mock or EV71 infection. The surviving EV71-infected and mock-infected cells were collected for sgRNA sequencing (Fig. S1 A). This screen identified 13 genes with multiple sgRNAs showing at least fivefold greater expression in EV71-infected cells compared with mock-infected cells (Fig. 1 A, left panel). These genes are as follows: (i) eight genes associated with enterovirus receptors, including the gene (*SCARB2*) encoding scavenger receptor class B member 2 and 7 genes (*XYLT2*, *B4GALT7*, *FAM20B*, *B3GALT6*, *B3GAT3*, *UGDH*, and *SLC35B2*) involved in the synthesis of sulfated glycosaminoglycans (sGAGs), an alternative glycan receptor for enteroviruses (Baggen et al., 2019; Nishimura et al., 2009; Yamayoshi et al., 2009); (ii) four genes (*COG2*, *COG3*, *COG5*, and *COG8*) encoding subunits of the COG complex which were known to be involved in enterovirus replication (Morosky et al., 2016); and (iii) a Ragulator component named *LAMTOR3* (Fig. 1 A, right panel).

To verify its role in EV71 infection, we used CRISPR-Cas9-mediated gene editing to knockout (KO) *LAMTOR3* in HeLa cells. Gene editing was verified by DNA sequencing and Western blotting (Fig. 1 B and Fig. S1 B). Compared with *LAMTOR3* wild-type HeLa cells, two *LAMTOR3*-KO clones had significantly increased cell viability after EV71 infection (Fig. 1 B). Reintroduction of *LAMTOR3* into *LAMTOR3*-KO HeLa cells restored virus-associated cell death (Fig. 1 C). Compared with mock-infected cells, EV71 infection increased the number of dying cells with damaged membranes, as indicated by propidium iodide (PI) staining, and the number of PI-positive cells was greatly reduced in EV7-infected *LAMTOR3*-KO cells compared with infected wild-type cells (Fig. 1 D). Consistently, re-expression of *LAMTOR3* in *LAMTOR3*-KO HeLa cells restored the percentage of PI-positive cells after EV71 infection to levels similar to infected wild-type cells (Fig. 1 D). The requirement for *LAMTOR3* in EV71-induced cell death was further confirmed in human rhabdomyosarcoma RD cells (Fig. 1 E), where deletion of *LAMTOR3* also efficiently reduced the percentage of PI-positive cells after EV71 infection (Fig. 1 F).

Because LAMTOR3 is a component of the Ragulator complex, we examined the roles of other Ragulator components in EV71-associated cell death. Like *LAMTOR3* deficiency, the deletion of *LAMTOR1* in two HeLa clones significantly increased cell viability and reduced the percentage of PI-positive cells (Fig. 1, G and H and Fig. S1 C). Moreover, RNAi-mediated silencing of *LAMTOR2* or *LAMTOR4* significantly inhibited EV71-associated cell death (Fig. S1, D–F). We further examined the role of Ragulator components on EV71-induced cytopathy by examining the activation of apoptosis and pyroptosis. Notably, loss of LAMTOR1 or LAMTOR3 inhibited the activation (cleavage) of caspase-9, caspase-3, and GSDME (Fig. 1, I and J). Collectively, these results demonstrate that loss of Ragulator components protects host cells against EV71 infection-induced apoptosis and pyroptosis.

### The Ragulator-Rag complex is required for EV71 replication

**Figure 2.**
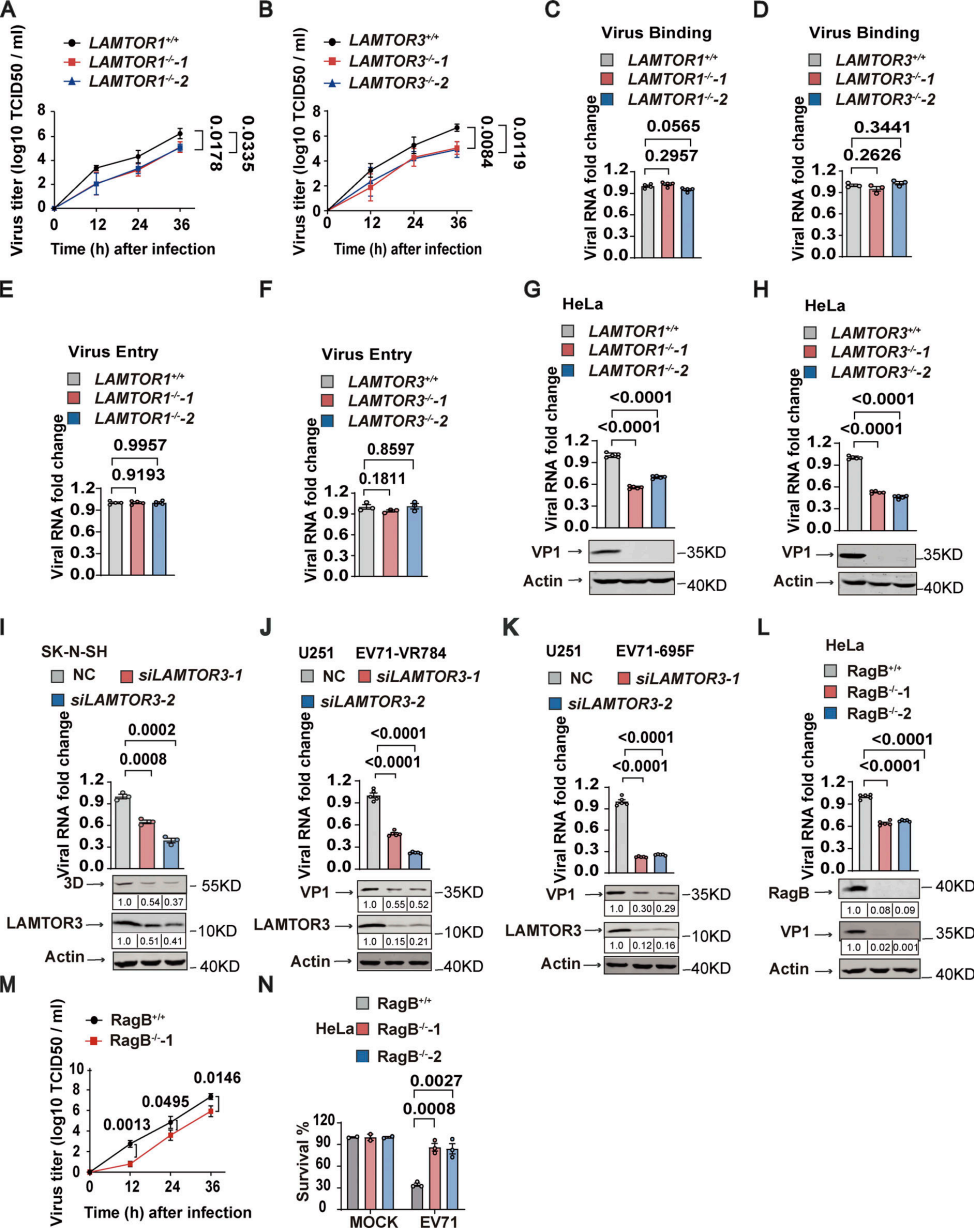
**The Ragulator-Rag complex is required for EV71 replication. (A and B)** Wild-type, *LAMTOR1*^*−/−*^ (A), and *LAMTOR3*^*−/−*^ (B) HeLa cells were infected with EV71 (MOI = 5) and the viral titers were determined by TCID50 assay at 12, 24, and 36 h after infection. **(C–F)** Wild-type, *LAMTOR1*^*−/−*^, and *LAMTOR3*^*−/−*^ HeLa cells were incubated with EV71 (MOI > 100) at 4°C for 1 h. For the virus binding assay, cells were washed with 1×PBS three times, then total cellular RNA was extracted and viral RNA was quantified by qPCR (C and D). For the virus entry assay, these cells were then transferred to a 37°C incubator for 20 min after 1 h of incubation at 4°C and were rinsed three times with 1×PBS-HCl. Total RNA was extracted, and viral RNA was quantified by qPCR (E and F). All values are means ± SEM. Data were analyzed using two-tailed Student’s *t* test. **(G and H)** Wild-type, *LAMTOR1*^*−/−*^ (G), and *LAMTOR3*^*−/−*^ (H) HeLa cells were infected with EV71 (MOI = 5). Viral RNA was quantified by qPCR 5 h after infection. VP1 expression was analyzed by Western blot 12 h after infection. All values are means ± SEM. Data were analyzed using two-tailed Student’s *t* test. **(I)** SK-N-SH cells were transfected with *LAMTOR3* siRNAs and then infected with EV71 (MOI = 5) after 60 h. The knockdown efficiency of *LAMTOR3* was determined by Western blotting. Viral RNA level was quantified by qPCR 5 h after infection. 3D expression was analyzed by Western blot 12 h after infection. The quantitation analysis of Western blot was performed using ImageJ. All values are means ± SEM. Data were analyzed using two-tailed Student’s *t* test. **(J and K)** U251 cells were transfected with *LAMTOR3* siRNAs and then infected with EV71-VR784 (MOI = 5; J) or EV71-695F (MOI = 5; K) after 60 h. Viral RNA was quantified by qPCR 5 h after infection. VP1 expression was analyzed by Western blot 12 h after infection. The quantitation analysis of Western blot was performed using ImageJ. All values are means ± SEM. Data were analyzed using two-tailed Student’s *t* test. **(L–N)** RagB^*+/+*^ and RagB^*−/−*^ HeLa cells were infected with EV71 (MOI = 5). Viral RNA was quantified by qPCR 5 h after infection. The knockout efficiency of RagB in HeLa cells and VP1 expression were detected by Western blot 12 h after infection (L). The quantitation analysis of Western blot was performed using ImageJ. Viral titers were determined by TCID50 assay at 12, 24, and 36 h after infection (M). Cell death induced by EV71 was detected by measuring ATP levels 18 h after infection (N). All values are means ± SEM. Data were analyzed using two-tailed Student’s *t* test. Source data are available for this figure: SourceData F2.

**Figure S2.**
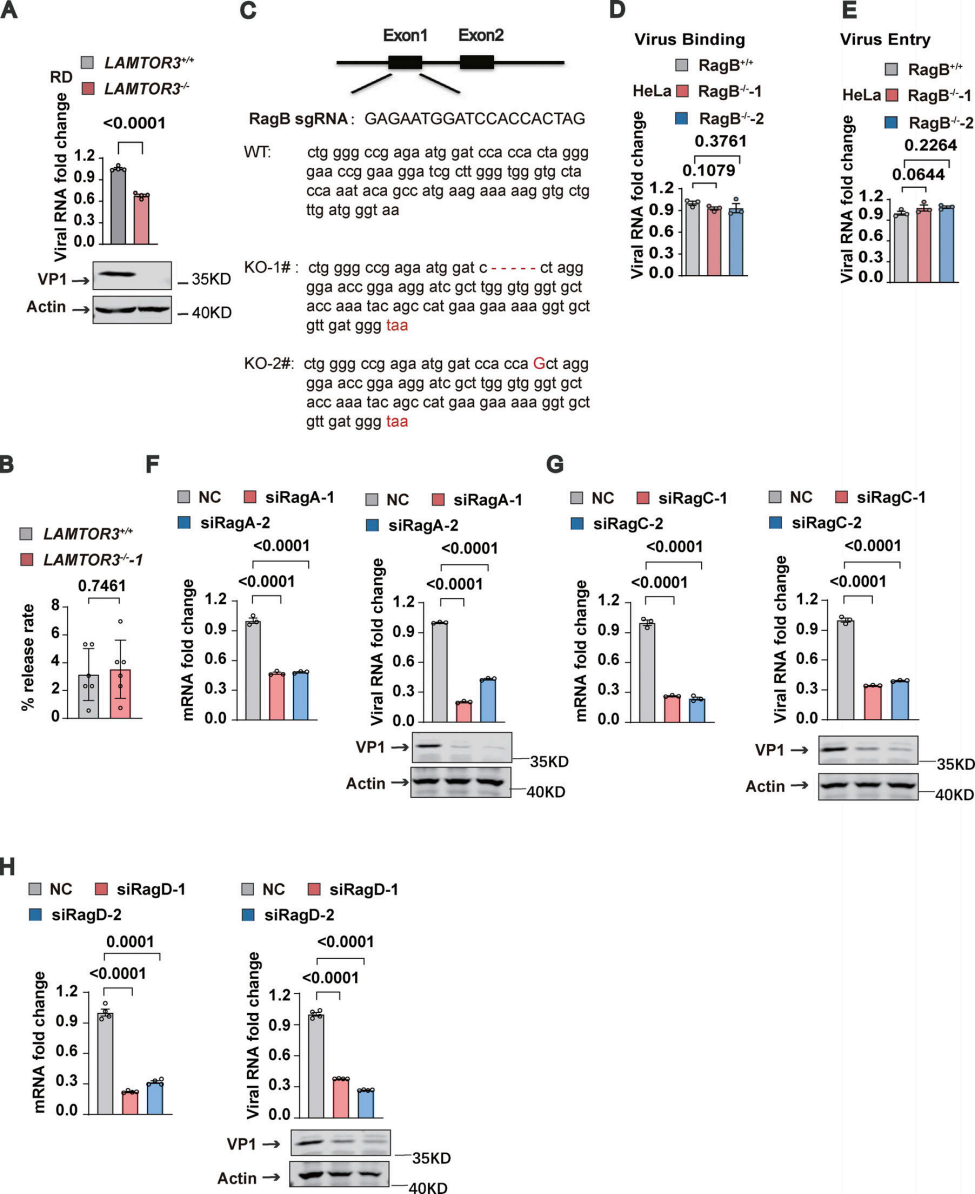
**The Ragulator-Rag complex is required for EV71 replication. (A)** *LAMTOR3*^*+/+*^ or *LAMTOR3*^*−/−*^ RD cells were infected with EV71 (MOI = 2). The viral RNA was quantified by qPCR 5 h after infection and the VP1 protein was analyzed by Western blot 8 h after EV71 infection. All values are means ± SEM. Data were analyzed using two-tailed Student’s *t* test. **(B)** WT and LAMTOR3-KO cells were infected with EV71 for 24 h. The virus titers in the culture supernatant and cells were measured by TCID50 assay. The release rate represents the proportion of virus in the supernatant relative to the total virus in WT cells and LAMTOR3-KO cells. All values are means ± SEM. Data were analyzed using two-tailed Student’s *t* test. **(C)** Sequencing analysis of knockout efficiency of RagB. **(D and E)** RagB^+/+^ and RagB^−/−^ HeLa cells were incubated with EV71 (MOI > 100) at 4°C for 1 h. For the virus binding assay, cells were washed with 1×PBS three times, then total cellular RNA was extracted and viral RNA was quantified by qPCR (D). For the virus entry assay, these cells were transferred to a 37°C incubator for 20 min after 1 h of incubation at 4°C and then rinsed three times with 1×PBS-HCl. Total RNA was extracted and viral RNA was quantified by qPCR (E). All values are means ± SEM. Data were analyzed using two-tailed Student’s *t* test. **(F–H)** *RagA*, *RagC*, and *RagD* were silenced in HeLa cells using siRNAs and then the indicated HeLa cells were infected with EV71 (MOI = 5) 60 h after transfection. The knockdown efficiency of *RagA* (F), *RagC* (G), and *RagD* (H) was determined by qPCR 60 h after transfection. Viral RNA (F–H) was quantified by qPCR 5 h after infection. VP1 expression (F–H) was analyzed by Western blot 12 h after infection. All values are means ± SEM. Data were analyzed using two-tailed Student’s *t* test. Source data are available for this figure: SourceData FS2.

We next examined the effects of Ragulator components on viral titers in EV71-infected cells. Both *LAMTOR1*-KO and *LAMTOR3*-KO HeLa cells had significantly reduced virus titers post EV71 infection compared with their wild-type counterparts (Fig. 2, A and B), suggesting a functional role for Ragulator in EV71 infection. To determine whether Ragulator components facilitate EV71 entry into the cell, we assessed virus binding and internalization. Loss of *LAMTOR1* or *LAMTOR3* had no obvious effect on EV71 binding to cells (Fig. 2, C and D), nor did loss of either gene prevent EV71 entry into cells (Fig. 2, E and F). However, both *LAMTOR1*-KO and *LAMTOR3*-KO HeLa cells showed significantly inhibited EV71 genome replication (Fig. 2, G and H). The requirement for LAMTOR3 in EV71 replication was further confirmed in human rhabdomyosarcoma RD cells, human neuroblastoma SK-N-SH cells, and human glioblastoma U251 cells, in which we observed significantly reduced viral RNA replication after *LAMTOR3* KO or knockdown (Fig. S2 A; and Fig. 2, I and J). *LAMTOR3* silencing also significantly inhibited viral replication of EV71-695F, a different EV71 strain (Zhang et al., 2020; Fig. 2 K). Moreover, knocking out *LAMTOR3* in HeLa cells or knocking down *LAMTOR3* in SK-N-SH cells and U251 cells strongly attenuated the expression of viral proteins such as VP1 and 3D (Fig. 2, G–K). To examine the impact of LAMTOR3 on EV71 virus release, we calculated the percentage of virus present in the supernatant compared to the total virus in both WT cells and LAMTOR3-KO cells. The result indicates that the absence of LAMTOR3 has no obvious effect on EV71 virus release (Fig. S2 B). Taken together, these results demonstrate the important role of Ragulator in EV71 replication.

Given that Ragulator interacts with Rag GTPases and acts as a guanylate exchange factor to activate Rag GTPases (Bar-Peled et al., 2012), we examined the role of Rag GTPases in EV71 infection. We knocked out RagB, a necessary component for the assembly of the Rag complex (Sancak et al., 2008), in HeLa cells (Fig. 2 L and Fig. S2 C) and found that RagB-KO cells had significantly decreased EV71 RNA replication and viral titers (Fig. 2, L and M). Moreover, EV71-associated cell death was inhibited in RagB-KO cells (Fig. 2 N). Like Ragulator, RagB had no effect on virus binding or the entry of EV71 into cells (Fig. S2, D and E). In addition, RNAi-mediated silencing of RagA, RagC, or RagD significantly inhibited EV71 RNA replication and the expression of EV71 VP-1 protein (Fig. S2, F–H). These results indicate that the Ragulator-Rag complex is required for EV71 replication.

### The Ragulator-Rag complex recruits EV71 3D protein and PI4KB

**Figure 3.**
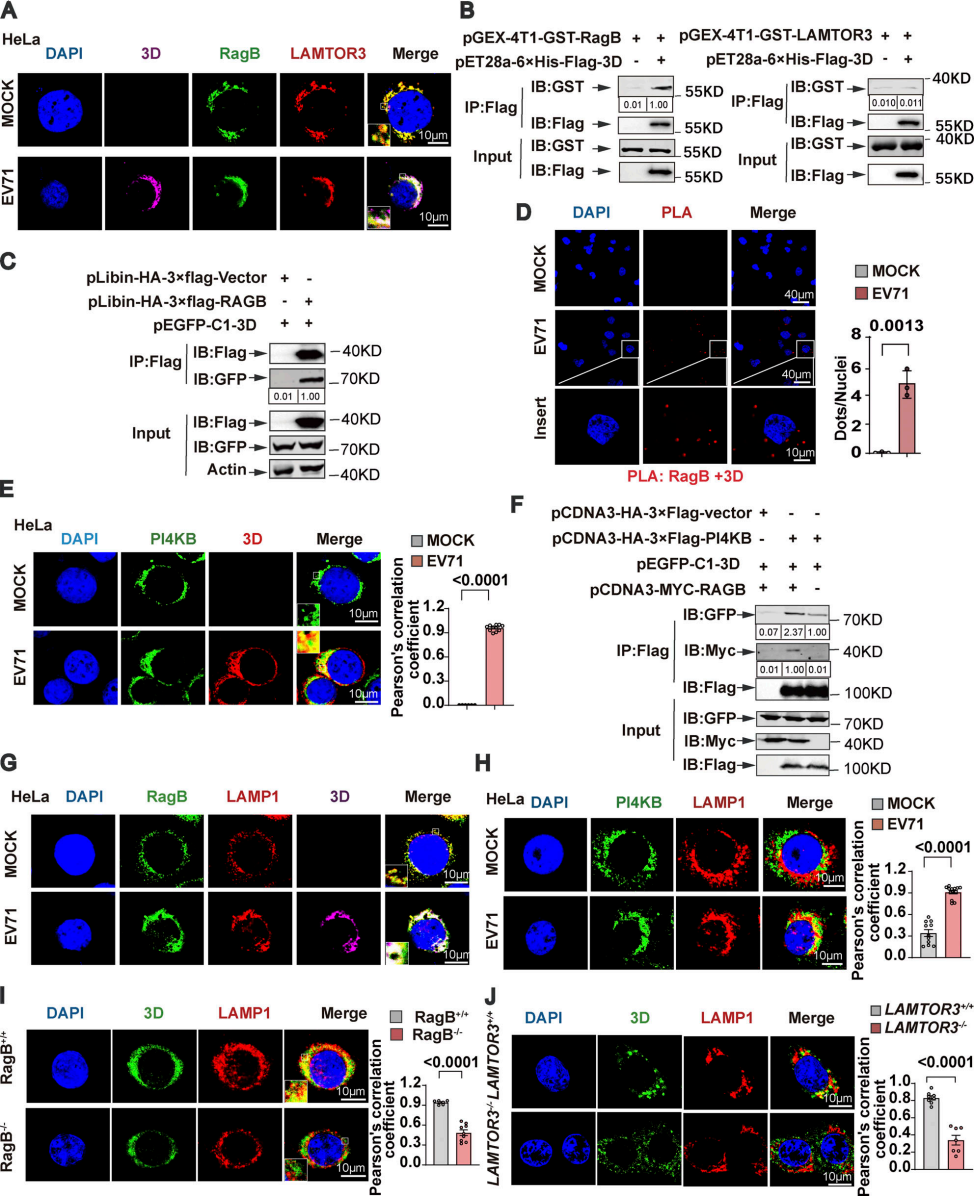
**The Ragulator-Rag complex recruits EV71 3D protein and PI4KB. (A)** HeLa cells were infected with EV71 (MOI = 5) for 6 h and then the colocalization of LAMTOR3, RagB, and 3D was examined by staining cells with anti-LAMTOR3/RagB/3D antibodies followed by confocal microscopy analysis. Nuclei were stained with DAPI. Scale bars, 10 μm. Inset panels are magnified 4×. **(B)** GST pull-down assay of in vitro translated Flag-tagged 3D and GST-tagged RagB/LAMTOR3. The quantitation analysis of Western blot was performed using ImageJ. **(C)** The plasmid encoding HA-3×FLAG-RagB and the plasmid encoding GFP-tagged 3D were cotransfected into 293T cells. Cell lysates were collected after 36 h for IP with anti-FLAG agarose and the interaction between 3D and RagB was detected by Western blotting. The quantitation analysis of Western blot was performed using ImageJ. **(D)** RD cells were harvested 6 h after EV71 infection and analyzed using the in situ PLA. Representative images of in situ PLA show the endogenous interaction between RagB and 3D (red). Nuclei were stained with DAPI. Scale bars, 10 and 40 μm. All values are means ± SEM. Data were analyzed using two-tailed Student’s *t* test. **(E)** HeLa cells were mock infected or infected with EV71 (MOI = 5) for 6 h. Colocalization of PI4KB and 3D was detected by staining cells with anti-PI4KB and anti-3D antibodies and visualizing by confocal microscopy. Nuclei were stained with DAPI. Scale bars, 10 μm. Inset panels are magnified 4×. The Pearson’s correlation coefficient of PI4KB and 3D was analyzed by Image pro plus 6.0 (right panel). All values are means ± SEM. Data were analyzed using two-tailed Student’s *t* test. **(F)** 293T cells were transfected with plasmids expressing HA-3×Flag-PI4KB, GFP-3D, and Myc-RagB, as indicated. Cell lysates were collected after 36 h and subjected to IP using anti-FLAG agarose beads. Interactions between PI4KB and 3D were detected by Western blot. The quantitation analysis of Western blot was performed using ImageJ. **(G)** HeLa cells were mock infected or infected with EV71 (MOI = 5) for 6 h. Colocalization of LAMP1, RagB, and 3D was assessed by staining cells with anti-LAMP1/RagB/3D antibodies followed by visualizing by confocal microscopy. Nuclei were stained with DAPI. Scale bars, 10 μm. Inset panels are magnified 4×. **(H)** HeLa cells were mock infected or infected with EV71 (MOI = 5) for 6 h. Colocalization of PI4KB and LAMP1 was assessed by staining cells with anti-PI4KB and anti-LAMP1 antibodies and visualizing by confocal microscopy. Nuclei were stained with DAPI. Scale bars, 10 μm. The Pearson's correlation coefficient of PI4KB and LAMP1 was analyzed by Image pro plus 6.0 (right panel). All values are means ± SEM. Data were analyzed using two-tailed Student’s *t* test. **(I and J)** Wild-type HeLa cells were infected with EV71 (MOI = 5). RagB^−/−^ (I) and *LAMTOR3*^*−/−*^ (J) HeLa cells were infected with EV71 (MOI = 20). Co-localization of 3D with LAMP1 was assessed by staining cells with anti-LAMP1 and anti-3D antibodies and visualizing by confocal microscopy. Nuclei were stained with DAPI. Scale bars, 10 μm. Inset panels are magnified 4×. The Pearson's correlation coefficient of 3D and LAMP1 was analyzed by Image pro plus 6.0 (right panel). All values are means ± SEM. Data were analyzed using two-tailed Student’s *t* test. Source data are available for this figure: SourceData F3.

**Figure S3.**
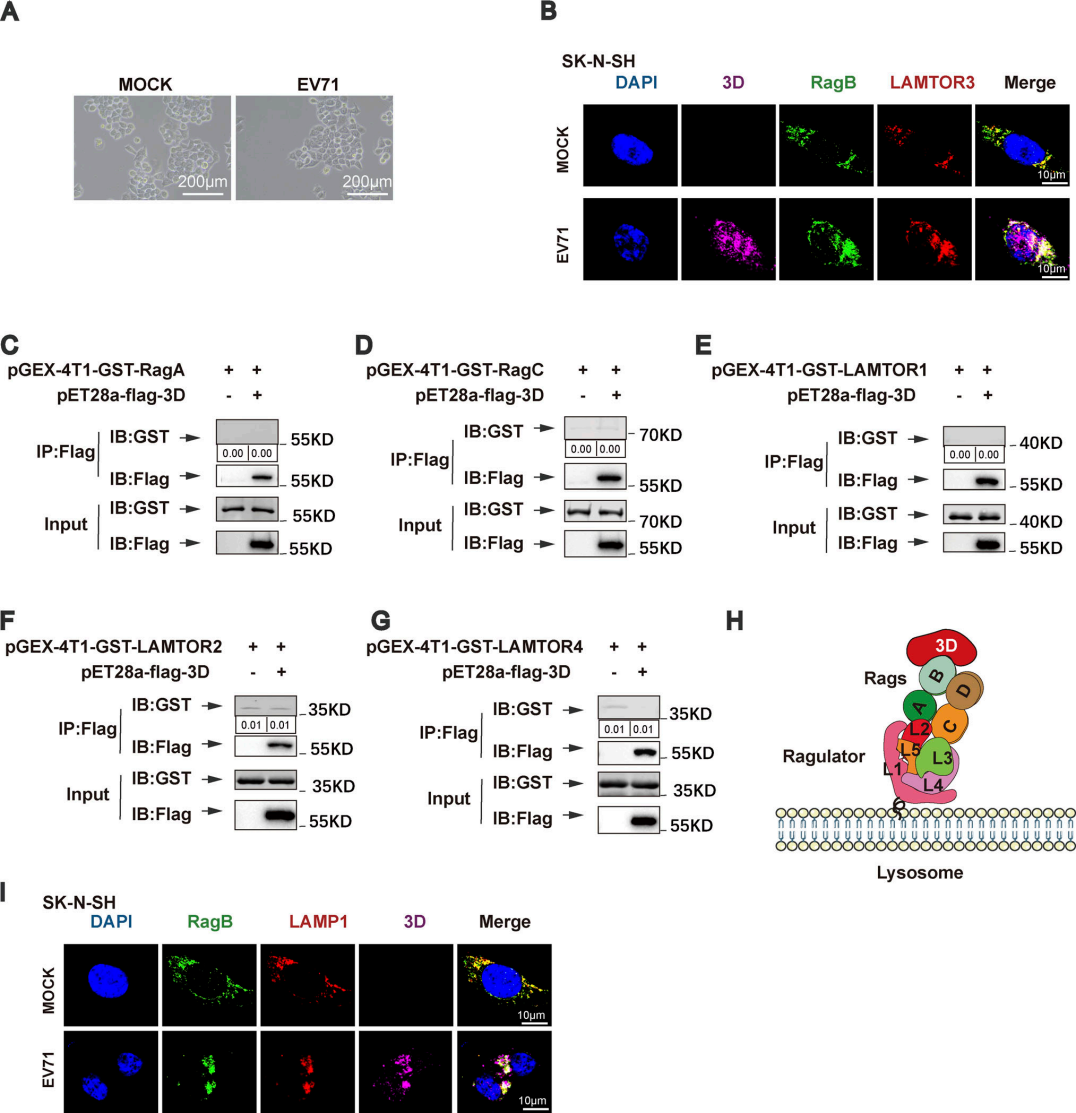
**The Ragulator-Rag complex recruits EV71 3D protein and PI4KB. (A)** HeLa cells were mock infected or infected with EV71 (MOI = 5) and the changes in cellular morphology were observed under a microscope at 6 h after infection. Scale bars, 200 μm. **(B)** SK-N-SH cells were mock infected or infected with EV71 (MOI = 5) for 12 h. Colocalization of RagB, LAMTOR3, and 3D was detected by staining cells with anti-RagB/LAMTOR3/3D antibodies and visualizing by confocal microscopy. Nuclei were stained with DAPI. Scale bars, 10 μm. **(C–G)** GST pull-down assay of in vitro translated Flag-tagged 3D and GST-tagged RagA, RagC, LAMTOR1, LAMTOR2, and LAMTOR4. The quantitation analysis of Western blot was performed using ImageJ. **(H)** Schematic diagram of EV71 3D protein binding to Ragulator-Rag complex. **(I)** SK-N-SH cells were mock-infected or infected with EV71 (MOI = 5) for 12 h. Colocalization of RagB, LAMP1, and 3D was detected by staining cells with anti-LAMP1/RagB/3D antibodies and visualizing by confocal microscopy. Nuclei were stained with DAPI. Scale bars, 10 μm. Source data are available for this figure: SourceData FS3.

To explore how Ragulator-Rag facilitates EV71 replication, we examined the association between the Ragulator-Rag complex and viral polymerase 3D, a key regulator of EV71 genome replication. In EV71-infected cells, 3D was enriched in the cytoplasm, and a fraction of 3D was colocalized with LAMTOR3 and RagB when no apparent cytopathic effect on cells was observed (Fig. 3 A; and Fig. S3, A and B). These results indicate a possible physical interaction between 3D and LAMTOR3 or RagB. Further, by using bacterially expressed recombinant proteins, we observed that 3D directly interacted with RagB, rather than with other components of the Ragulator-Rag complex (Fig. 3 B and Fig. S3, C–H). The interaction between 3D and RagB was further confirmed by precipitating a FLAG-RagB immunocomplex (Fig. 3 C). Moreover, in situ proximity ligation assay (PLA) revealed the formation of a 3D-RagB complex in EV71-infected RD cells (Fig. 3 D). 3D is known to interact with PI4KB (Hsu et al., 2010; Zhang et al., 2021). Consistently, we observed colocalization of PI4KB with 3D in EV71-infected cells (Fig. 3 E), and overexpression of RagB enhanced the interaction between PI4KB and 3D (Fig. 3 F). Collectively, these results support that EV71 3D and PI4KB are recruited to the Ragulator-Rag complex in EV71-infected cells.

Previous studies have shown that Ragulator tethers Rags to the surface of lysosomes (Sancak et al., 2010). We examined the subcellular localization of the RagB-3D complex and found that in both EV71-infected HeLa and SK-N-SH cells, 3D and RagB colocalized with the lysosomal membrane protein, LAMP1 (Fig. 3 G and Fig. S3 I). Moreover, PI4KB also colocalized with LAMP1 in EV71-infected HeLa cells (Fig. 3 H). Of note, the localization of 3D on the lysosomal surface was diminished in RagB-KO cells (Fig. 3 I), indicating that RagB is required for the localization of 3D on the lysosomal membrane. Finally, we demonstrated that the deletion of *LAMTOR3* also blocked the localization of 3D on the lysosomal surface (Fig. 3 J). Collectively, these results demonstrate that the Ragulator-Rag complex recruits EV71 3D protein and PI4KB to the lysosomal surface.

### Disrupting lysosomal tethering of the Ragulator-Rag-3D complex inhibits EV71 replication

**Figure 4.**
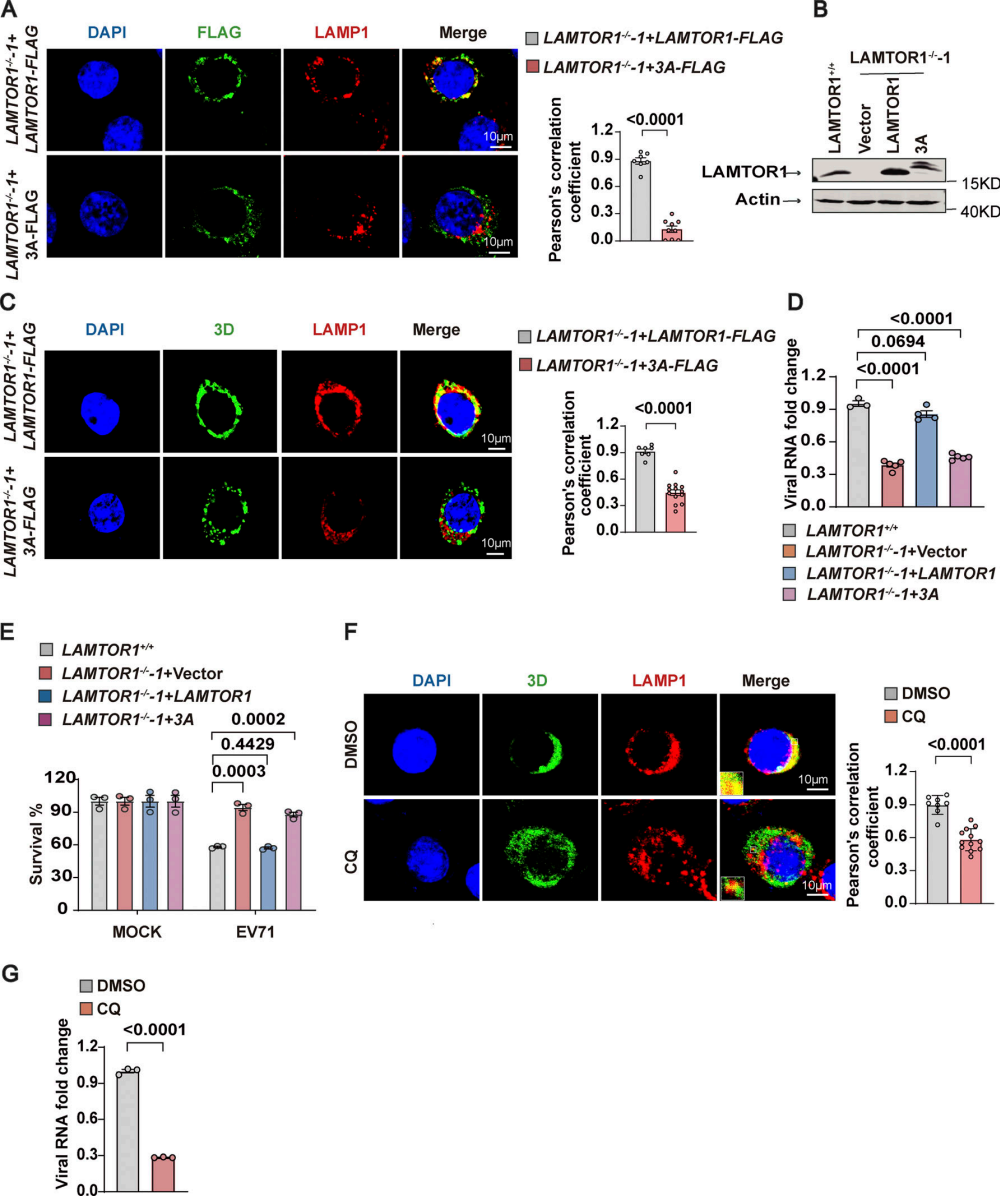
**Disrupting lysosomal tethering of the Ragulator**-**Rag-3D complex inhibits EV71 replication. (A)** Co-localization of LAMTOR1 with LAMP1 was assessed by confocal microscopy in indicated HeLa cells (*LAMTOR1*^*−/−*^-1+*LAMTOR1*-FLAG and *LAMTOR1*^*−/−*^-1+ *LAMTOR1*-3A-FLAG). FLAG (green), LAMP1 (red), and DAPI-stained nuclei are shown. Scale bars, 10 μm (left panel). The Pearson’s correlation coefficient of FLAG and LAMP1 were analyzed by Image pro plus 6.0 (right panel). All values are means ± SEM. Data were analyzed using two-tailed Student’s *t* test. **(B)** Western blot analysis of the ectopic expression of *LAMTOR1* and *LAMTOR1*-3A in *LAMTOR1*^*−/−*^ cells. **(C)** *LAMTOR1*^*−/−*^-1+*LAMTOR1*-FLAG and *LAMTOR1*^*−/−*^-1+ *LAMTOR1*-3A-FLAG HeLa cells were infected with EV71 (MOI = 5 and MOI = 40, respectively) for 6 h. Co-localization of 3D with LAMP1 was assessed by staining cells with anti-LAMP1 and anti-3D antibodies followed by visualizing by confocal microscopy. Nuclei were stained with DAPI. Scale bars, 10 μm (left panel). The Pearson’s correlation coefficient of 3D and LAMP1 was analyzed by Image pro plus 6.0 (right panel). All values are means ± SEM. Data were analyzed using two-tailed Student’s *t* test. **(D and E)** The indicated HeLa cells were mock infected or infected with EV71 (MOI = 5). Viral RNA was detected by qPCR 5 h after infection (D). Cell viability was determined by measuring ATP levels 24 h after infection (E). All values are means ± SEM. Data were analyzed using two-tailed Student’s *t* test. **(F)** RD cells were pretreated with DMSO or CQ for 2 h and infected with EV71 (MOI = 5 for DMSO group; MOI = 10 for CQ group) for 6 h. The colocalization of 3D with LAMP1 was assessed by staining cells with anti-LAMP1 and anti-3D antibodies and visualizing by confocal microscopy. Nuclei were stained with DAPI. Scale bars, 10 μm (left panel). Inset panels are magnified 4×. The Pearson’s correlation coefficient of 3D and LAMP1 was analyzed by Image pro plus 6.0 (right panel). All values are means ± SEM. Data were analyzed using two-tailed Student’s *t* test. **(G)** RD cells were pretreated with DMSO or CQ for 2 h and infected with EV71. Viral RNA was quantified by qPCR 5 h after infection. All values are means ± SEM. Data were analyzed using two-tailed Student’s *t* test. Source data are available for this figure: SourceData F4.

Ragulator anchors to lysosomes via the G2 and C3C4 amino acid residues at the N-terminus of LAMTOR1 (Nada et al., 2009). Thus, to determine whether lysosomal tethering of Ragulator-Rag-3D plays a role in EV71 replication, we engineered *LAMTOR1*-KO HeLa cells to stably express a mutant form of LAMTOR1 in which G2, C3, and C4 were mutated to alanine (HeLa-LAMTOR1-3A). As expected, colocalization of LAMTOR1 with LAMP1 was diminished in HeLa-LAMTOR1-3A cells (Fig. 4, A and B). Moreover, in HeLa-LAMTOR-3A cells infected with EV71, 3D also failed to localize with LAMP1 (Fig. 4 C), indicating that the mutant LAMTOR1-3A disrupted 3D localization to the lysosomal surface. Importantly, we also found that replication of EV71 RNA and EV71-associated cell death were significantly reduced in HeLa-LAMTOR1-3A cells compared with infected HeLa cells with wild-type LAMTOR1 (Fig. 4, D and E). These results suggest that Ragulator-Rag complex-mediated recruitment of 3D to the lysosomal surface is important for EV71 replication.

The lysosomotropic drug, chloroquine (CQ), has been shown to inhibit HFMD-associated enteroviruses, including EV-71 (Lin et al., 2013; Tan et al., 2018). We therefore examined the effect of CQ on the subcellular localization of EV71 3D. Pretreatment of CQ for 2 h reduced the localization of 3D to the lysosomal surface as well as reduced viral replication of EV71 (Fig. 4, F and G), further supporting that disrupting lysosomal tethering of EV71 3D suppresses viral replication.

### The lysosomal tethered Ragulator-Rag complex mediates CVA16 replication

**Figure 5.**
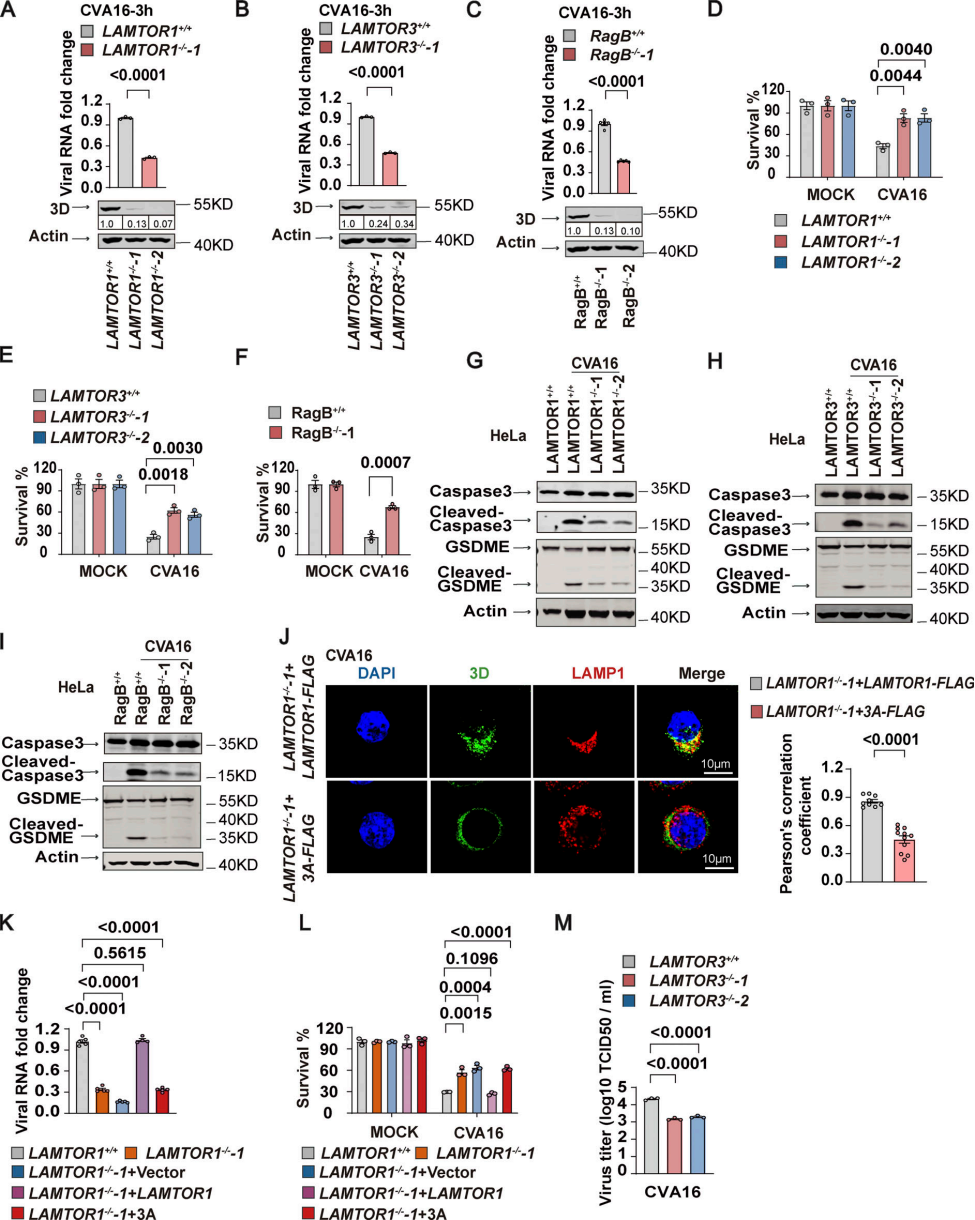
**The lysosomal tethered Ragulator-Rag complex mediates CVA16 replication. (A–C)** Wild-type, *LAMTOR1*^*−/−*^-1 (A), *LAMTOR3*^*−/−*^-1 (B), and RagB^*−/−*^-1 (C) HeLa cells were infected with CVA16 (MOI = 5). Viral RNA was quantified by qPCR 3 h after infection (upper panel). Expression of viral 3D was detected by Western blot analysis 12 h after infection (lower panel). The quantitation analysis of Western blot was performed using ImageJ. All values are means ± SEM. Data were analyzed using two-tailed Student’s *t* test. **(D–F)** Wild-type, *LAMTOR1*^*−/−*^-1 (D), *LAMTOR3*^*−/−*^-1 (E), and RagB^*−/−*^-1 (F) HeLa cells were infected with CVA16 (MOI = 5). Cell viability was determined by measuring ATP levels 24 h after infection. All values are means ± SEM. Data were analyzed using two-tailed Student’s *t* test. **(G–I)** Wild-type, *LAMTOR1*^*−/−*^-1 (G), *LAMTOR3*^*−/−*^-1 (H), and *RagB*^*−/−*^*-*1(I) HeLa cells were infected with CVA16 (MOI = 5). Cleavage of GSDME and caspase3 were analyzed by Western blot 12 h after infection. **(J)** *LAMTOR1*^*−/−*^-1+*LAMTOR1*-FLAG and *LAMTOR1*^*−/−*^-1+3A-FLAG HeLa cells were infected with CVA16 (MOI = 5 and MOI = 20, respectively). Colocalization of 3D with LAMP1 was detected by staining cells with anti-LAMP1 and anti-3D antibodies and visualizing by confocal microscopy. Nuclei were stained with DAPI. Scale bars, 10 μm (left panel). The Pearson’s correlation coefficient of 3D and LAMP1 was analyzed by Image pro plus 6.0 (right panel). All values are means ± SEM. Data were analyzed using two-tailed Student’s *t* test. **(K and L)** HeLa cells as indicated were infected with CVA16 (MOI = 5). Viral RNA was quantified by qPCR 5 h after infection (K). Cell viability was detected by measuring ATP levels 24 h after infection (L). All values are means ± SEM. Data were analyzed using two-tailed Student’s *t* test. **(M)** *LAMTOR3*^*+/+*^, *LAMTOR3*^*−/−*^-1, and *LAMTOR3*^*−/−*^-2 HeLa cells were infected with CVA16 (MOI = 5) and viral titers were determined by TCID50 assay 24 h after infection. All values are means ± SEM. Data were analyzed using two-tailed Student’s *t* test. Source data are available for this figure: SourceData F5.

Having shown that Ragulator-Rag is required for EV71 replication, we next examined the role of the Ragulator-Rag complex on CVA16 replication. Loss of either LAMTOR1 or LAMTOR3 significantly inhibited viral RNA replication in HeLa cells after CVA16 infection (Fig. 5, A and B, upper panel), and replication of CVA16 RNA was significantly decreased in RagB-KO cells (Fig. 5 C, upper panel). Consistently, deficiency of the Ragulator-Rag complex resulted in lower expression of CVA16 3D (Fig. 5, A–C, lower panel). Moreover, CVA16-mediated apoptosis and pyroptosis were significantly reduced in *LAMTOR1*-KO, *LAMTOR3*-KO, and RagB-KO HeLa cells compared with their wild-type counterparts (Fig. 5, D–I).

**Figure S4.**
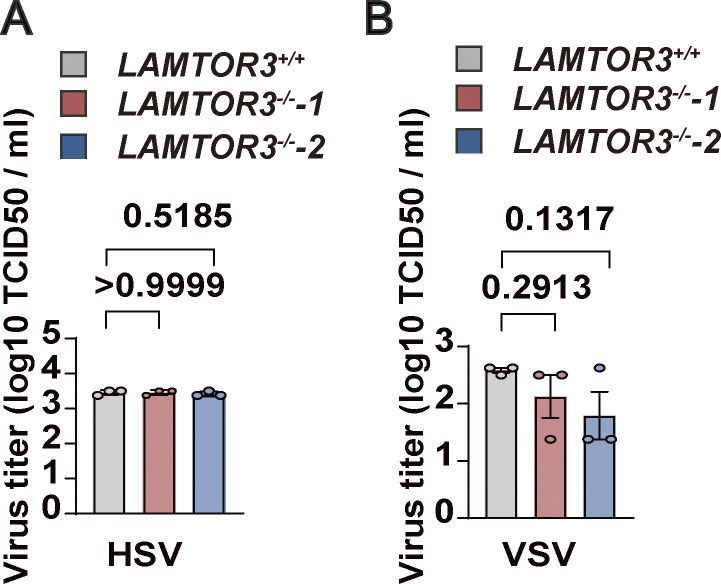
**The lysosomal tethered Ragulator-Rag complex mediates CVA16 replication.** (**A and B)** *LAMTOR3*^*+/+*^ and *LAMTOR3*^−/−^-1, *LAMTOR3*^−/−^-2 HeLa cells were infected with HSV (MOI = 2; A) or VSV (MOI = 0.5; B) and viral titers were determined by TCID50 assay 24 h after infection. All values are means ± SEM. Data were analyzed using two-tailed Student’s *t* test.

Because Ragulator-Rag recruits EV71 3D to the lysosomal surface for viral replication, we next assessed the subcellular localization of CVA16 3D. Like EV71 3D, CVA16 3D was localized on the surface of lysosomes (Fig. 5 J), and disruption of lysosomal tethering of Ragulator-Rag in HeLa-LAMTOR1-3A cells markedly inhibited the localization of CVA16 3D on the lysosomal surface (Fig. 5 J). Importantly, replication of CVA16 RNA, as well as virus-associated cell death, was also significantly blocked in HeLa-LAMTOR1-3A cells (Fig. 5, K and L). Of note, loss of LAMTOR3 significantly lowered the viral titer of CVA16 (Fig. 5 M), whereas it did not affect the viral titer of the non-enteroviruses viruses, herpes simplex virus (HSV), and vesicular stomatitis virus (VSV; Fig. S4, A and B). Thus, our results indicate that the Ragulator-Rag-3D axis mediates the replication of HFMD-causing enteroviruses, including EV71 and CVA16.

### ZHSI-1 is a novel EV71 inhibitor that interacts with 3D and blocks replication of EV71 and CVA16

**Figure 6.**
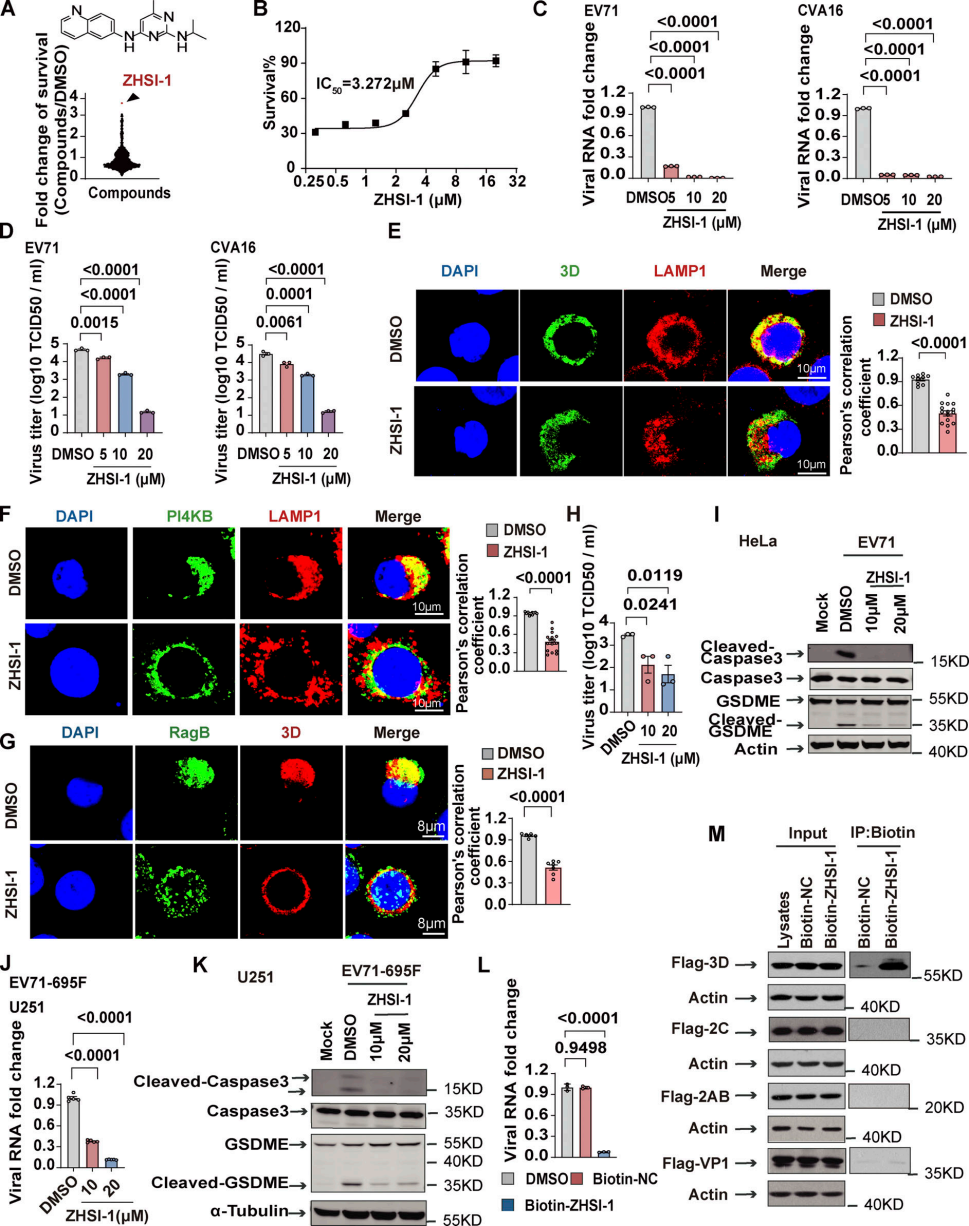
**ZHSI-1 is a novel EV71 inhibitor that interacts with 3D and blocks the replication of EV71 and CVA16. (A)** ZHSI-1 inhibits EV71-induced cell death. The schematic shows the chemical structure of ZHSI-1. **(B)** RD cells were pretreated with the indicated concentrations of ZHSI-1 and then infected with EV71 (MOI = 2). Cell viability was determined by measuring ATP levels 24 h after infection. **(C and D)** RD cells were pretreated with ZHSI-1 for 1 h and then infected with EV71 (MOI = 2) or CVA16 (MOI = 2). Viral RNA was quantified by qPCR 5 h after infection (C) and viral titers were determined by TCID50 assay 24 h after infection (D). All values are means ± SEM. Data were analyzed using two-tailed Student’s *t* test. **(E)** HeLa cells were infected with EV71 (MOI = 5) and then treated with ZHSI-1 at 5 μM 2 h after infection. Co-localization of LAMP1 and 3D was determined by staining cells with anti-LAMP1 and anti-3D antibodies and visualizing by confocal microscopy 7 h after infection. Nuclei were stained with DAPI. Scale bars, 10 μm. The Pearson’s correlation coefficient of 3D and LAMP1 was analyzed by Image pro plus 6.0 (right panel). All values are means ± SEM. Data were analyzed using two-tailed Student’s *t* test. **(F)** HeLa cells were infected with EV71 (MOI = 5) and then treated with ZHSI-1 at 5 μM 1 h after infection. Colocalization of LAMP1 and PI4KB was determined by staining cells with anti-LAMP1 and anti-PI4KB antibodies and visualizing by confocal microscopy 7 h after infection. Nuclei were stained with DAPI. Scale bars, 10 μm. The Pearson’s correlation coefficient of PI4KB and LAMP1 was analyzed by Image pro plus 6.0 (right panel). All values are means ± SEM. Data were analyzed using two-tailed Student’s *t* test. **(G)** HeLa cells were infected with EV71 (MOI = 5) and then treated with ZHSI-1 at 5 μM 2 h after infection. Colocalization of RagB and 3D was determined by staining cells with anti-RagB and anti-3D antibodies and visualizing by confocal microscopy 6 h after infection. The Pearson’s correlation coefficient of RagB and 3D was analyzed by Image pro plus 6.0 (right panel). Nuclei were stained with DAPI. Scale bars, 8 μm. All values are means ± SEM. Data were analyzed using two-tailed Student’s *t* test. **(H and I)** EV71-infected (MOI = 5) HeLa cells were treated with ZHSI-1 2 h after infection and viral titers were determined by TCID50 assay 24 h after infection (H). Cleavage of GSDME and caspase3 were analyzed by Western blot 12 h after infection (I). All values are means ± SEM. Data were analyzed using two-tailed Student’s *t* test. **(J and K)** EV71-695F-infected (MOI = 5) U251 cells were treated with ZHSI-1 2 h after infection. Viral RNA was quantified by qPCR 6 h after infection (J). Cleavage of GSDME and caspase3 were analyzed by Western blot 12 h after infection (K). All values are means ± SEM. Data were analyzed using two-tailed Student’s *t* test. **(L)** RD cells were pretreated with Biotin-ZHSI-1 or Biotin-NC for 1 h and then RD cells were infected with EV71 (MOI = 2). Viral RNA was quantified by qPCR 8 h after infection. All values are means ± SEM. Data were analyzed using two-tailed Student’s *t* test. **(M)** 293T cells were transfected with plasmids encoding FLAG-tagged VP1, 2AB, 2C, or 3D. After 48 h, cells were lysed and protein was isolated. Biotin-ZHSI-1 or Biotin-NC (negative control) was preincubated with avidin magnetic beads for 2 h and then incubated with the protein lysates overnight. Western blot analysis was used to detect viral proteins that bound to Biotin-ZHSI-1. Source data are available for this figure: SourceData F6.

**Figure S5.**
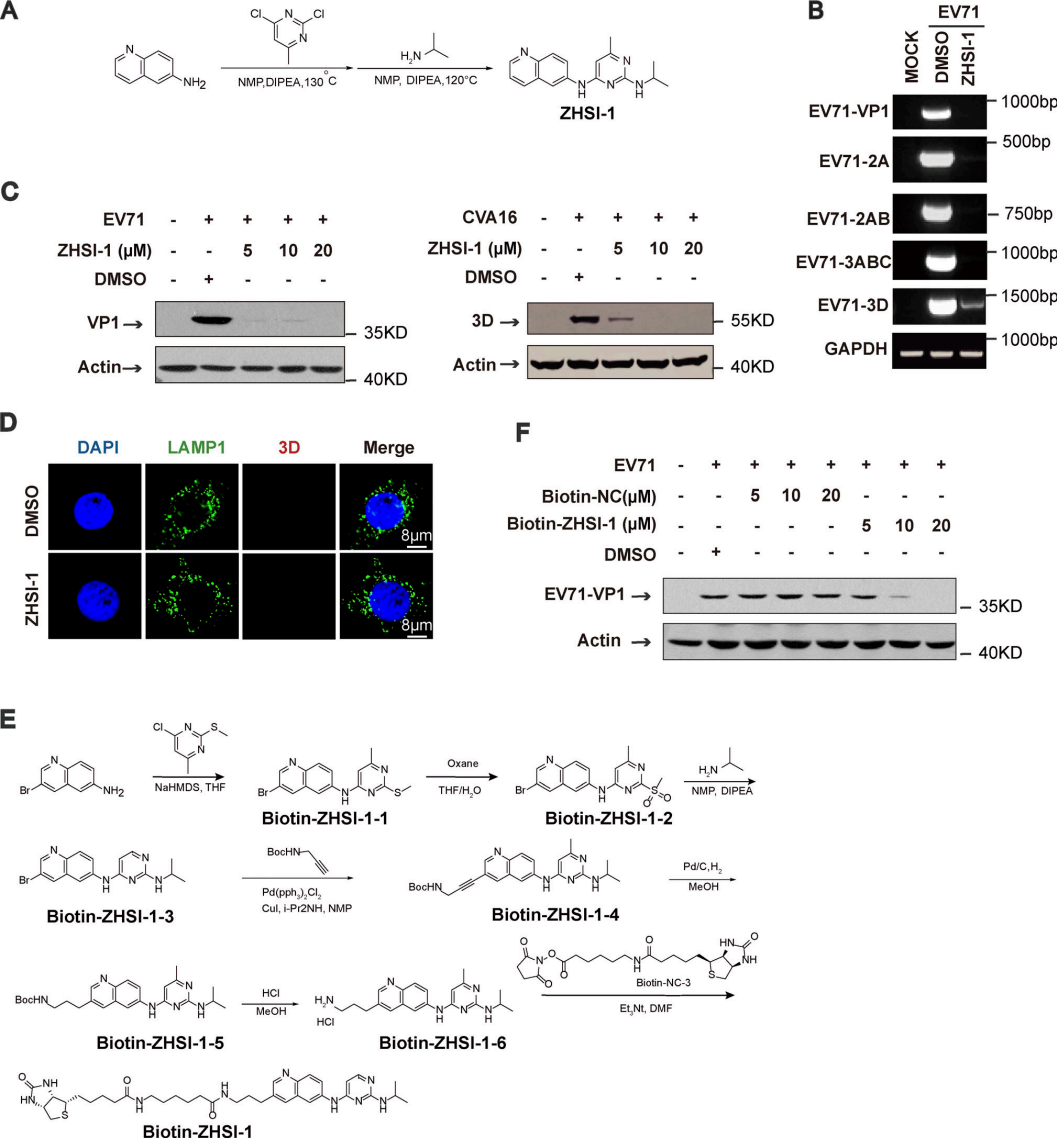
**ZHSI-1 is a novel EV71 inhibitor that interacts with 3D and blocks replication of EV71 and CVA16. (A)** The synthesis route for ZHSI-1. **(B)** RD cells were pretreated with ZHSI-1 for 1 h and then infected with EV71 (MOI = 2) for 8 h. EV71 RNA was quantified by semi-quantitative PCR. **(C)** RD cells were pretreated with ZHSI-1 for 1 h and then infected with EV71 (MOI = 5) or CVA16 (MOI = 5). Western blot analysis was performed to detect the expression of 3D or VP1 12 h after infection. **(D)** HeLa cells were treated with ZHSI-1 at 5 μM for 6 h. Cells were stained with anti-LAMP1 antibody and visualized by confocal microscopy. Nuclei were stained with DAPI. Scale bars, 8 μm. **(E)** The synthesis route for Biotin-ZHSI-1. **(F)** RD cells were pretreated with Biotin-ZHSI-1 or Biotin-NC for 1 h and then infected with EV71 (MOI = 2) for 8 h. The expression of VP1 was detected by Western blot analysis. Source data are available for this figure: SourceData FS5.

Our results so far indicate that the Ragulator-Rag-3D axis is a potential therapeutic target in HFMD caused by either EV71 or CVA16 infection. To identify novel inhibitors of EV71, we first screened a small-molecule library of ∼1,450 compounds to identify those that block EV71-induced cell death, followed by a second screen for compounds that interfere with lysosomal tethering of 3D. Among prioritized hits, ZHSI-1 most potently inhibited EV71-induced cell death with IC_50_ = 3.272 µM (Fig. 6, A and B; and Fig. S5 A). In infected RD cells, ZHSI-1 significantly reduced EV71 and CVA16 RNA replication and viral titers in a dose-dependent manner (Fig. 6, C and D), as well as strongly suppressed the expression of both EV71 genes and proteins compared with control-treated cells (Fig. S5, B and C). ZHSI-1 significantly inhibited the localization of 3D and PI4KB on the lysosomal surface (Fig. 6, E and F; and Fig. S5 D) and inhibited the colocalization of 3D and RagB (Fig. 6 G). Notably, even when ZHSI-1 treatment was not started until 2 h after infection with EV71, the compound still significantly reduced viral titers (Fig. 6 H). Treatment of EV71-infected cells with ZHSI-1 also inhibited EV71-induced pyroptosis (Fig. 6 I), and the inhibitory effect of ZHSI-1 on EV71 replication and virus-associated pyroptosis was further confirmed in experiments using U251 cells infected with the EV71-695F strain (Fig. 6, J and K).

To test whether ZHSI-1 directly targets 3D, we added a biotin tag to ZHSI-1 (Biotin-ZHSI-1) by chemical synthesis to allow precipitation of ZHSI-1-interacting proteins using streptavidin-conjugated beads (Fig. S5 E), and we confirmed that Biotin-ZHSI-1 retained the ability to inhibit EV71 replication (Fig. 6 L and Fig. S5 F). We also used a biotin-labeled negative control compound (Biotin-NC) that lacked the ability to inhibit EV71 replication (Fig. 6 L and Fig. S5 F). A co-IP assay with Biotin-ZHSI-1 indicated direct interaction between ZHSI-1-Biotin and 3D, but not with other viral proteins, including VP1, 2AB, and 2C (Fig. 6 M), suggesting 3D is a target of ZHSI-1.

### ZHSI-1 inhibits EV71 replication and viral pathogenesis in vivo

**Figure 7.**
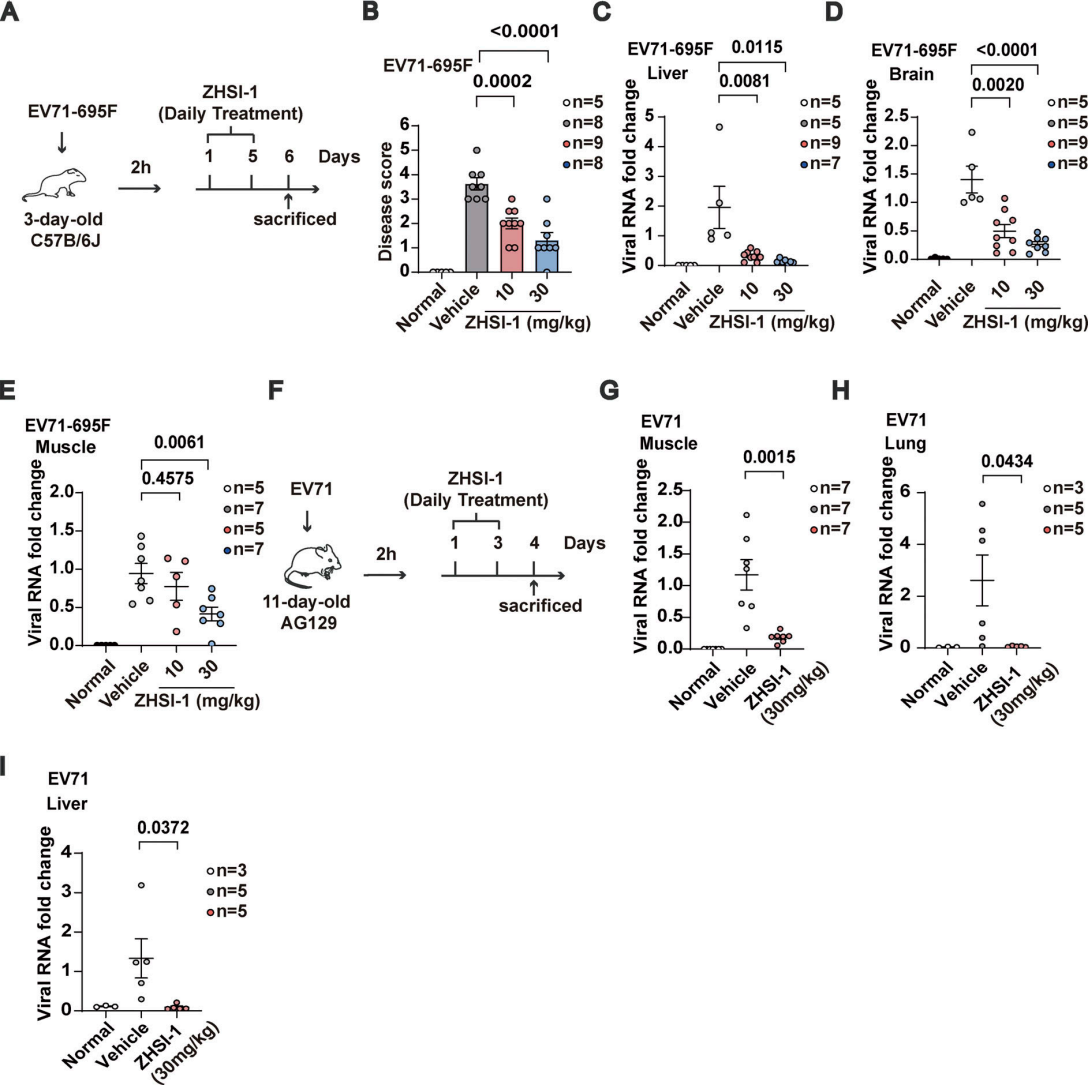
**ZHSI-1 inhibits EV71 replication and viral pathogenesis in vivo. (A)** Schematic diagram of the mouse model of EV71-695F infection. **(B–E)** 3-d-old C57BL/6J mice were injected intraperitoneally with EV71-695F or PBS and administered intraperitoneally with indicated concentrations of ZHSI-1-HCl 2 h after infection. Symptoms of paralysis were scored on day 6 (B). Clinical disease was scored as follows: 0, healthy; 1, ruffled hair and hunchbacked appearance; 2, limb weakness; 3, paralysis in one limb; 4, paralysis in two limbs; and 5, death. To minimize animal suffering, mice were euthanized if they were paralyzed in two limbs. Postmortem tissue was collected on day 6 and processed and viral RNA was quantified by qPCR (C–E). All values are means ± SEM. Data were analyzed using two-tailed Student’s *t* test. **(F)** Schematic diagram for the mouse model of EV71 (VR-784) infection. **(G–I)** 11-d-old AG129 mice were injected intraperitoneally with EV71 (VR-784) or PBS and administered intraperitoneally with ZHSI-1-HCl 2 h after infection. Postmortem tissue was collected on day 4 and processed and viral RNA was quantified by qPCR. All values are means ± SEM. Data were analyzed using two-tailed Student’s *t* test.

We next evaluated the therapeutic potential of ZHSI-1 against EV71 infection in vivo. EV71-695F can infect neonatal immune-competent mice (Zhang et al., 2020); thus, we selected neonatal C57 mice and infected them with EV71-695F for 2 h before randomizing the animals to treatment with vehicle or ZHSI-1 via intraperitoneal (i.p.) injection (Fig. 7 A). All vehicle-treated mice displayed symptoms of limb paralysis on day 6 after EV71-695F infection. However, treatment with ZHSI-1 significantly alleviated the EV71-695F-induced paralysis phenotype in EV71-695F-infected mice in a dose-dependent manner (Fig. 7 B). Consistently, treatment with ZHSI-1 efficiently reduced the levels of EV71-695F RNA in the liver, brain, and muscles of mice (Fig. 7, C–E). The anti-EV71 activity of ZHSI-1 was further confirmed in a similar study of EV71 infection in 11-d-old AG129 mice, which are deficient in interferon-α/β and γ receptors and have been used for EV71 infection (Khong et al., 2012; van den Broek et al., 1995). In vehicle-treated mice, high expression levels of viral RNA were detected in the muscles, liver, and lungs, but viral RNA levels were markedly reduced in these tissues in ZHSI-1-treated animals (Fig. 7, F–I). Collectively, our results indicate that targeting 3D with ZHSI-1 is effective in inhibiting EV71 infection.

## Discussion

Our study demonstrates that the lysosome-tethered Ragulator-Rag complex is required for the replication of EV71 and CVA16. Upon EV71 or CVA16 infection, the Ragulator-Rag complex recruits viral 3D to the lysosomal surface through the binding of 3D to RagB. Disruption of the lysosomal tethering of Ragulator-Rag significantly reduces the replication of EV71 and CVA16. We also discovered ZHSI-1, a novel inhibitor of EV71/CVA16 that targets 3D. In virus-infected cells, ZHSI-1 blocks lysosomal tethering of 3D and markedly inhibits replication of viral RNA as well as pyroptosis. In vivo, treatment with ZHSI-1 effectively protected both neonatal and young mice against EV71 infection. Our study indicates targeting the lysosome-tethered Ragulator-Rag-3D complex may be a promising antiviral strategy for treating EV71/CVA16-associated HFMD.

Enteroviruses induce the formation of ROs, which are membrane-bound structures that facilitate viral RNA replication (Baggen et al., 2018; Hsu et al., 2010). Studies have shown that the ROs of EV71 are associated with the Golgi or endoplasmic reticulum and comprise host factors such as PI4KB as well as viral proteins, including 3A and 3D (Hsu et al., 2010; Laufman et al., 2019). 3A recruits PI4KB to the replication sites by interacting with the acyl-CoA binding domain containing 3 (ACBD3; Lyoo et al., 2019; Xiao et al., 2017). Then, PI4KB catalyzes the formation of a PI4P-rich microenvironment conducive to the recruitment of 3D (Hsu et al., 2010; Xiao et al., 2017). Based on a genome-wide CRISPR-Cas9 knockout screen, we identified the host factor, Ragulator, as a mediator for EV71 replication. We observed colocalization of 3D with LAMTOR3, RagB, and the lysosomal marker LAMP1 during EV71 infection. Using the biochemical and PLA approaches, we also observed the interaction between 3D and RagB. The absence of LAMTOR3 or RagB in EV71-infected cells prevented colocalization of 3D with LAMP1, suggesting that 3D is recruited to the lysosome by the lysosome-tethered Ragulator-Rag complex. Studies have shown that CQ has inhibitory effects on EV71 entry and EV71 replication (Tan et al., 2018; Wang et al., 2020). We observed increased influence intensity of LAMP1 and RagB after EV71 infection (Fig. 3 G). To enhance the expression of 3D protein in the CQ-treated cells, we added a higher MOI of EV71 into the cells treated with CQ compared with the control group (Fig. 4 F). We noted a decrease in the colocalization of 3D with LAMP1 in the CQ-treated cells. While this result suggests that CQ reduces the colocalization of 3D with LAMP1, we cannot definitively rule out the impact of CQ on viral entry. PI4KB can also be recruited to lysosome-tethered Ragulator-Rag upon EV71 infection; moreover, RagB promotes the association between 3D and PI4KB. Since 3D and PI4KB are crucial components of ROs during EV71/CVA16 replication, accumulation of these components at the Ragulator-Rag complex positioned on the lysosome membrane may potentially serve as viral replication sites or aid in the formation of ROs for EV71/CVA16.

The Ragulator-Rag complex plays a critical role in the regulation of nutrient-sensing and metabolic pathways (Bar-Peled and Sabatini, 2014; Yonehara et al., 2017). Ragulator-Rag is anchored to lysosomes and serves as an activator of mTORC1, a master regulator of cell growth and metabolism (Saxton and Sabatini, 2017). Enteroviruses, like EV71 and CVA16, require the biogenesis pathways of the host cell to generate the lipids and proteins necessary to optimize their replication (Baggen et al., 2018; Hsu et al., 2010; McPhail et al., 2020; Xiao et al., 2017). We established a functional role of Ragulator-Rag in mediating viral RNA replication for both EV71 and CVA16, whereas Ragulator-Rag did not affect viral binding or entry into cells. Given the importance of the Ragulator-Rag complex in metabolic pathways, the 3D recruitment to the lysosomal tethering Ragulator-Rag complex could potentially coordinate cellular biogenesis for EV71/CVA16 replication.

HFMD is considered a significant public health threat to infants and young children due to the lack of specific antiviral drugs approved for its treatment (Lim et al., 2020). Although there has been progress toward the development of antiviral drugs against EV71 by targeting viral proteins or host factors (Lim et al., 2020; Yan et al., 2022), these are not yet clinically available, and there remains an urgent need to develop effective therapies. We found that ZHSI-1 binds to EV71 3D and reduces the recruitment of 3D to lysosome-tethered Ragulator-Rag. Treatment of EV71- or CVA16-infected cells with ZHSI-1 effectively inhibited viral replication. Previous studies have shown that EV71 infection induces pyroptosis in both neuronal and non-neuronal cells (Dong et al., 2022). Deficiency of the pyroptosis mediator, GSDME, attenuates neurological symptoms in mice challenged with EV71 infection (Dong et al., 2022). We showed that treatment with ZHSI-1 significantly inhibited GSDME-mediated pyroptosis induced by EV-71 infection. Remarkably, treatment with ZHSI-1 also effectively inhibited viral RNA replication and improved clinical symptoms in young mice with EV71 infection. Therefore, our study indicates that targeting 3D with ZHSI-1 may hold promise for treating HFMD caused by EV71 and CVA16 infection and may be effective against HFMD caused by other viruses as well. Future studies will be required to understand the precise mechanism by which ZHSI-1 interacts with 3D polymerase to modulate its function; such studies are expected to increase our understanding of the biology of enterovirus replication as well as inform optimized strategies for antiviral drugs.

## Materials and methods

### Cell lines and viruses

HeLa, human rhabdomyosarcoma RD cells, human neuroblastoma SK-N-SH cells, human glioblastoma U251 cells, and HEK293T cells were obtained from the American Type Culture Collection (ATCC). SK-N-SH cells were cultured in MEM (C11095500BT; Gibco) containing 10% fetal bovine serum (FBS; Sunrise) and 100 U/ml of penicillin and streptomycin (10378016; Gibco). The other cells were cultured in DMEM (C11995500BT; Gibco) containing 10% FBS and 100 U/ml of penicillin and streptomycin. All cells were maintained in a humidified incubator with 5% CO_2_ at 37°C.

EV71 strain VR-784 was purchased from China Center for Type Culture Collection (CCTCC). EV71 strain SHAPHC695F/SH/CHN/10 (EV71-695F; GenBank: JQ736684.2) was isolated from fecal sample of a 1.8-yr-old patient in Shanghai public health clinical centre (SHPHC) in 2010 and provided by Dr. Zhigang Yi (Fudan University, Shanghai, China; Zhang et al., 2013). Coxsackievirus A16 (CVA16-G10; GeneBank: U05876.1) was provided by Dr. Dan Xu (Fudan University, Shanghai, China). VSV was from Genhong Cheng Laboratory (University of California, Los Angeles, CA, USA). HSV virus was kindly provided by Dr. Chunfu Zheng (Soochow University, Suzhou, China). Viral titers were determined using the TCID50 assay according to standard procedures.

### Mice

C57BL/6J (B6) mice were purchased from Beijing Vital River. AG129 mice were purchased from Marshall BioResources. All mice were housed in a pathogen-free animal facility at Suzhou Institute of Systems Medicine. All animal experiments were performed in accordance with protocols approved by the Animal Care and Use Committee of the Suzhou Institute of Systems Medicine.

### Plasmid constructs

To generate the N-terminal HA-3×FLAG tag and N-terminal Myc tag gene-expressing construct, the empty pCDNA3 vector was redesigned by inserting the HA-3×Flag or Myc tag sequence into the vector. To construct plasmids expressing *LAMTOR3*, RagB, and EV71 3D, fragments of *LAMTOR3*, RagB, and EV71 3D cDNA were cloned into the pCDNA3 vector engineered with the HA-3×Flag tag or Myc tag. All constructs were verified by DNA sequencing.

### Genome-wide CRISPR-Cas9 screens

The lentiviral gRNA plasmid library used for genome-wide CRISPR-Cas9 screening was purchased from Addgene (#1000000095). Amplification of the library was performed according to the manufacturer’s protocol provided by Addgene. Genome-wide CRISPR-Cas9 screening was carried out as previously described (Shi et al., 2015). In brief, HEK293T cells were transfected with 25 μg library DNA, 15 μg psPAX2, and 10 μg pMD2.G and cultured in 15-cm dishes. The cell supernatant was harvested and stored at −80°C at 24 and 48 h after transfection. HeLa cells overexpressing Cas9 were seeded in a 15-cm cell culture dish at a density of 3 × 10^6^. A total of 3 × 10^7^ HeLa-Cas9 cells were seeded in 10 15-cm cell culture dishes and infected with the lentivirus library. Puromycin was added 72 h after infection with lentivirus. 7 d after puromycin addition, nine culture dishes were treated with EV71 to trigger cell death; one dish was left untreated as a control sample. 36 h after EV71 infection, the supernatant was replaced with a fresh complete DMEM medium. Surviving cells were collected after 15 d. Genomic DNA was prepared from each group of cells for sequencing.

The lentiviral gRNA plasmid library contains a total of 187,536 sgRNAs targeting 18,543 genes. Each gene is targeted by 10 sgRNAs with different sequences. After sequencing, the abundance of at least three sgRNAs from a gene that shows a more than fivefold increase was selected as a potential hit for further examination.

### Knockout cell lines

All sgRNA sequences were derived from Addgene. The sequence of sgRNA targeting *LAMTOR3* was 5′-TTG​CAA​CAG​ACC​AAG​GAA​GC-3′; the sequence of sgRNA targeting *LAMTOR1* was 5′-TGG​GGT​GCT​GCT​ACA​GCA​GC-3′; and the sequence of sgRNA targeting RagB was 5′-GAG​AAT​GGA​TCC​ACC​ACT​AG-3′. In brief, the sgRNAs were cloned into the sgRNA-Cas9-expressing plasmid, pX458-GFP (Addgene). To construct the KO cell lines, 1 μg of PX458-GFP-sgRNA was transfected into HeLa cells in a six-well plate. After 72 h, the cells were sorted by GFP fluorescence. KO efficiency was verified by sequencing and Western blotting.

### Cell viability assay and PI staining

Cells were seeded in 96-well plates and then infected with EV71 (multiplicity of infection [MOI] = 1) or CVA16 (MOI = 1) after 24 h. For cell viability assays, cell viability was measured 18–24 h after viral infection using the Cell Titer-Glo Luminescent Cell Viability Assay kit (Promega) according to the manufacturer’s instructions. Luminescence was calculated with SpectraMax i3x (Molecular Devices). For PI staining, 5 μl PI was added to the 96-well plate 18–24 h after viral infection and fluorescence was captured with a fluorescence microscope.

### Gene silencing

Small-interfering RNAs (siRNAs) were used to silence *LAMTOR2*, *LAMTOR3*, *LAMTOR4*, *RagA*, *RagC*, and *RagD* in cells. The sequences for oligos targeting *LAMTOR2*, *LAMTOR3*, and *LAMTOR4* have been described previously (Zhang et al., 2014). The sequences for oligos targeting *RagA*, *RagC*, and *RagD* were purchased from Qiagen. Knockdown efficiency was verified by q-PCR. In brief, Lipofectamine 2000 (11668030; Invitrogen) or INTERFERin (101000028; Polyplus) was used to transfect 25–40 nm oligos into cells according to the manufacturer’s instructions. After 60 h of transfection, cells were infected with EV71 (MOI = 0.5 or 5). Cell viability assay and Western blotting were performed on transfected cells.

### RNA extraction, reverse transcription, and quantitative PCR

RNA was extracted using Trizol Reagent (Invitrogen) according to the manufacturer’s instructions. For each sample, 1 μg RNA was reverse transcribed into cDNA using HiScript II Q RT SuperMix (Vazyme). For quantitative PCR (qPCR) assays, SYBR Green Master Mix (B21202; Bimake) was used to detect gene expression using a Roche LightCycler 480 II system. The primer sequences are listed in Table S1.

### Protein lysate preparation, Western blotting, and quantification of immunoblot signals

Cell pellets were harvested by centrifugation at 10,000 *g* for 1 min and then resuspended in lysis buffer (20 mM Tris-HCl pH 7.4, 150 mM NaCl, 10% glycerol, 1% Triton X-100, 1 mM Na_3_VO_4_, 25 mM β-glycerol phosphate, 0.1 mM PMSF, complete protease inhibitor cocktail). The cell lysates were placed on ice for 30 min and then centrifuged at 13,000 *g* for 30 min at 4°C. The supernatants were collected and proteins were transferred to PVDF membranes. Primary antibodies were diluted in PBST with 5% milk and incubated at 4°C overnight. After washing, membranes were incubated with anti-rabbit or anti-mouse IgG secondary antibody in PBST with 5% milk for 1 h at RT. Gel images were captured using the 800 nm signal channel of an Odyssey CLx infrared imaging system (LI-COR). For the detection of FLAG-tag, signals were acquired using the chemiluminescence horseradish peroxidase (HRP) substrate (Millipore). For the quantification of immunoblot signals, the intensities of each band were measured using ImageJ from inverted, 8-bit images. Antibodies were used as follows: LAMTOR3 (Cat #8168; Cell Signaling Technology), LAMTOR1 (Cat #8975S; Cell Signaling Technology), RagB (Cat #8150S, RRID:AB_11178806; Cell Signaling Technology), Enterovirus 71 (VP1; Cat #ab169442; Abcam), Enterovirus 71 3D (Cat #GTX630193, RRID:AB_2888196; GeneTex), GSDME (Cat #ab215191; Abcam), Cas9 (Cat #ab191468; Abcam), Caspase-3 (Cat #9662s; Cell Signaling Technology), Cleaved-Caspase-3 (Cat #9661; Cell Signaling Technology), FLAG-tag (Cat #A8592; Sigma-Aldrich), Caspase-9 (Cat #9508, RRID:AB_2068620; Cell Signaling Technology), Myc-tag (Cat #16286-1-AP, RRID:AB_11182162; Proteintech), β-actin (Cat #A2066; Sigma-Aldrich) anti-mouse IgG secondary antibody (Cat #92632210; LI-COR Biosciences), and anti-rabbit IgG secondary antibody (Cat #926-32211, RRID:AB_621843; LI-COR Biosciences).

### Immunoprecipitation

Plasmids expressing PI4KB and 3D with or without RagB were cotransfected into 293T cells in 6-cm plates. 36 h after transfection, these cells were collected and lysed with the cell lysis buffer. The lysed cells were incubated on ice for 30 min, followed by centrifuging at 13,000 *g* for 20 min at 4°C. An aliquot of the supernatant was retained for analysis as input, and the remaining cell lysates were incubated with anti-FLAG M2 agarose beads (Sigma-Aldrich) at 4°C with rocking for 16 h. The beads were washed eight times with lysis buffer. The precipitated proteins were eluted from beads using 2× SDS loading buffer at 95°C for 10 min. Western blotting was performed to identify interacting proteins.

### GST pull-down

GST-tagged proteins and 6×His-flag-tagged proteins were produced in BL21 Competent *Escherichia coli*. The GST-tagged proteins were purified using glutathione-sepharose 4B beads (17075601; Cytiva), and 6×His-flag-tagged proteins were purified by using Ni-NTA Agarose (R10-22-40-42/43; Qiagen). In vitro GST pull-down assay, the recombinant GST-tagged proteins were incubated with 6×His-flag-3D in buffer A (20 mM Tris, pH8.0; 50 mM NaCl; 1 mM EDTA; 1 mM DTT) and then incubated with anti-FLAG M2 agarose beads (Sigma-Aldrich) at 4°C with rocking for 12 h. The beads were washed eight times with buffer A. The precipitated proteins were eluted from beads using 2× SDS loading buffer at 95°C for 10 min and Western blotting was performed to identify interacting proteins.

### Virus titration

Viral titer was determined by using the TCID50 assay. Briefly, wild-type HeLa cells or HeLa cells deficient for LAMTOR1, LAMTOR3, or RagB were infected with EV71 (MOI = 5). Then, cells were harvested at 12, 24, and 36 h after EV71 infection and stored at −80°C. The virus supernatants were diluted 10-fold in PBS and incubated with RD cells in a 96-well plate. The cytopathic effect (CPE) on RD cells was observed after 72 h. The TCID50 was calculated using the Spearman & Kärber method.

### Immunofluorescence staining

HeLa cells were infected with EV71 for 6 h and then fixed with 4% paraformaldehyde (PFA) for 20 min and washed three times with 1×PBS followed by permeabilizing with 0.3% Triton X-100 for 20 min. Immunofluorescence confocal microscopy was performed using a TSA Signal Amplification kit (NECC4100; Histova Biotechnology) according to the manufacturer’s instructions. Briefly, the samples were blocked with blocking buffer for 1 h and then incubated with primary antibody overnight. Cells were washed three times with 1×PBS and incubated with secondary antibody for 30 min at 37°C, followed by incubation with fluorescence-conjugated TSA substrate. Confocal images were then taken using a Leica TCS SP8 MP.

### In situ proximity ligation assay

RD cells were infected with EV71 (MOI = 5) for 6 h, fixed with 4% PFA for 20 min, washed three times with 1×PBS, and then permeabilized with Triton X-100 for 20 min. The PLA assay was performed as previously described (Yu et al., 2023). In brief, cells were blocked at room temperature for 1 h with blocking solution (DUO82007) and then incubated with a primary antibody in dilution buffer (mouse anti-3D antibody: 1:100; rabbit anti-RagB: 1:75) overnight at 4°C. Next, the cells were subjected to the PLA assay (Cat #DUO92008; Sigma-Aldrich) with anti-mouse (Cat #DUO92001, RRID: AB_2810939; Sigma-Aldrich) and anti-rabbit (Cat #DUO92005, RRID: AB_2810942; Sigma-Aldrich) secondary antibodies following the manufacturer’s instructions. Nuclei were stained with DAPI. Confocal images were taken using a Leica TCS SP8 MP.

### Virus binding and internalization assays

For virus binding and internalization assays, 1 × 10^5^ cells were seeded in a 12-well plate and cultured for 24 h. The next day, these cells were infected with EV71 (MOI > 100) for 1 h. To determine EV71 binding, cells were washed three times with cold PBS and then collected, and viral RNA was detected by qPCR. To determine the internalization of the virus, cells were incubated at 37°C in prewarmed DMEM for 1 h after EV71 infection. After 10 min, cells were rinsed three times with 1×PBS-HCl (pH 1.3) to remove viral particles that were adsorbed on the cell surface but did not enter the cells. Finally, these cells were collected and viral RNA was detected by qPCR.

### Compounds screening against EV71 infection

RD cells were pretreated with small molecule compounds for 1 h and infected with EV71. After 24 h of infection, cell viability was measured using the CTG assay. Cycloheximide (CHX) was used as a positive control in the screening. We selected compounds that significantly inhibited EV71-induced cell death and exhibited a stronger inhibitory effect than CHX as candidates. ZHSI-1 was the most effective compound in inhibiting EV71-induced cell death. For a second screen, HeLa cells were infected with EV71 for 2 h and then treated with candidates. After 6 h of infection, cells were collected and fixed with 4% PFA, followed by the immunofluorescence experiment for analysis of the colocalization of 3D and LAMP1. ZHSI-1 showed inhibition of the colocalization of 3D and LAMP1.

### Mouse model of EV71 infection

EV71 infection in mice was carried out as previously described (Khong et al., 2012; Zhang et al., 2020) . 3-d-old suckling C57BL/6J mice (2.0–2.2 g) were randomly assigned to different groups. Mice received i.p. injection with 1.8 × 10^4^ pfu/mouse of EV71-695F diluted in 25 μl PBS. Mock-infected mice were injected with 25 μl PBS (Zhang et al., 2020). 2 h after infection, mice were treated with different dosages of ZHSI-1 (10 or 30 mg/kg) daily for 5 d. Clinical disease was scored on day 6 as follows: 0, healthy; 1, ruffled hair and hunchbacked appearance; 2, limb weakness; 3, paralysis in one limb; 4, paralysis in two limbs; and 5, death. To minimize animal suffering, mice were euthanized if they were paralyzed in two limbs. The mice were sacrificed on the sixth day after infection. Mouse tissues were collected and processed and the levels of viral RNA were detected by qPCR. To minimize animal suffering, mice were euthanized if they were paralyzed in two limbs before the study endpoint.

11-d-old AG129 mice received i.p. injection with 1 × 10^7^ pfu/mouse of EV71 diluted in 25 μl PBS (Khong et al., 2012). 2 h after infection, mice were treated daily with ZHSI-1 (30 mg/kg) for 3 d and euthanized on the fourth day. Tissue collection and analysis, and indications for euthanasia were the same as aforementioned.

### Detailed synthetic procedure for ZHSI-1 and biotin-ZHSI-1

General reaction progress was monitored by analytical thin-layer chromatography performed on silica gel HSGF254 precoated plates. Organic solutions were dried over anhydrous Na_2_SO_4_ and the solvents were removed under reduced pressure. The final compounds were purified with silica gel 100–300 mesh for column chromatography. ^1^H NMR was performed using a 400 MHz (Varian) spectrometer or 300 MHz (Vnmrs) spectrometer. Chemical shifts were presented as parts per million (ppm) using tetramethylsilane as an internal standard. Mass spectra were obtained using an Agilent 1100 LC/MSD Trap SL version Mass Spectrometer.

#### ZHSI-1: *N*^*2*^-isopropyl-6-methyl-*N*^*4*^-(quinolin-6-yl) pyrimidine-2,4-diamine

DIPEA (194 mg, 1.5 mmol) was added to a solution of quinolin-6-amine (160 mg, 1.1 mmol) and 2,4-dichloro-6-methylpyrimidine (200 mg, 1.2 mmol) in NMP (15 ml). The mixture was stirred at 130°C overnight. After cooling to room temperature, propan-2-amine (729 mg, 12 mmol) and DIPEA (194 mg, 1.5 mmol) were added and the mixture was stirred at 120°C overnight. The solvent was diluted with ethyl acetate (50 ml) and the organic layer was washed with saturated NaCl aqueous solution (20 ml × 5), dried over Na_2_SO_4_, and concentrated. The residue was purified by silica gel column chromatography (dichloromethane/methane = 20/1) to produce the final compound, ZHSI-1, which is a yellow solid (20 mg, 6.2%). ^1^H NMR spectrum: (400 MHz, CDCl_3_) δ 8.84 (s, 1H), 8.14 (s, 1H), 8.11–8.02 (m, 2H), 7.68–7.59 (m, 1H), 7.40 (s, 1H), 7.26 (s, 1H), 7.09 (s, 1H), 5.91 (s, 1H), 4.24–4.06 (m, 1H), 2.25 (s, 3H), 1.30 (s, 6H).

#### Biotin-ZHSI-1-1: 3-bromo-*N*-(6-methyl-2-(methylthio) pyrimidin-4-yl) quinolin-6-amine

First, 2 M NaHMDS (3.0 ml, 6.0 mmol) was added to a solution of 3-bromoquinolin-6-amine (669 mg, 3.0 mmol) and 4-chloro-6-methyl-2-(methylthio) pyrimidine (524 mg, 3.0 mmol) in dry THF (5 ml) under N_2_ atmosphere at 0°C. The mixture was stirred at room temperature for 2 h. Saturated NaHCO_3_ aqueous solution (5 ml) was added to quench the solution. The aqueous solution was extracted by ethyl acetate (10 ml × 3). The organic layer was combined, dried over Na_2_SO_4_, and concentrated. The residue was purified by silica gel column chromatography (ethyl acetate) to give the desired compound Biotin-ZHSI-1-1, which is a yellow solid. ^1^H NMR spectrum: (400 MHz, CDCl_3_) δ 8.81 (d, *J* = 2.4 Hz, 1H), 8.23 (d, *J* = 2.0 Hz, 1H), 8.04 (d, *J* = 9.2 Hz, 1H), 8.01 (d, *J* = 2.4 Hz, 1H), 7.64–7.62 (m, 1H), 6.89 (s, 1H), 6.31 (s, 1H), 2.60 (s, 3H), 2.36 (s, 3H).

#### Biotin-ZHSI-1-2: 3-bromo-*N*-(6-methyl-2-(methylsulfonyl) pyrimidin-4-yl) quinolin-6-amine

Oxone (636 mg, 2.0 mmol) was added to a solution of intermediate Biotin-ZHSI-1-1 (361 mg, 1.0 mmol) in THF/H_2_O (3/0.15 ml). The mixture was stirred at room temperature overnight. The solvent was removed to give the crude product Biotin-ZHSI-1-2 (1.0 g), which is a yellow solid.

#### Biotin-ZHSI-1-3: *N*^*4*^-(3-Bromoquinolin-6-yl)-*N*^*2*^-isopropyl-6-methylpyrimidine-2,4-diamine

DIPEA (645 mg, 5.0 mmol) and propan-2-amine (590 mg, 10.0 mmol) were added to a solution of crude intermediate Biotin-ZHSI-1-2 (1.0 g, 1.0 mmol) in NMP (5 ml). The mixture was stirred at 100°C overnight. After cooling to room temperature, the solvent was poured into ice water (20 ml). The resulting solid was filtered, washed with Et_2_O, and purified by silica gel column chromatography (ethyl acetate) to give the desired compound Biotin-ZHSI-1-3, which is a yellow solid (223 mg, 60%). ^1^H NMR spectrum: (400 MHz, CDCl_3_) δ 8.78 (d, *J* = 1.6 Hz, 1H), 8.20 (s, 1 H), 8.11 (s, 1H), 8.00 (d, *J* = 9.2 Hz, 1H), 7.62 (dd, *J* = 9.2, 1.6 Hz, 1H), 6.66 (s, 1H), 5.95 (s, 1H), 4.86 (d, *J* = 6.4 Hz, 1H), 4.25–4.12 (m, 1H), 2.25 (s, 3H), 1.29 (d, *J* = 5.6 Hz, 6H).

#### Biotin-ZHSI-1-4: Tert-butyl(3-(6-((2-(isopropylamino)-6-methylpyrimidin-4-yl)amino) quinolin-3-yl)prop-2-yn-1-yl)carbamate

Tert-butyl prop-2-yn-1-ylcarbamate (93 mg, 0.6 mmol), propan-2-amine (1 ml), Pd(dppf)Cl_2_ (7 mg, 0.01 mmol), and CuI (1 mg, 0.005 mmol) were added to a solution of Biotin-ZHSI-1-3 (100 mg, 0.27 mmol) in NMP (2 ml). The mixture was stirred under N_2_ atmosphere at 100°C for 20 h. The solvent was filtered, and the filtrate was poured into water (10 ml). The aqueous layer was extracted by ethyl acetate (10 ml × 3). The organic layers were combined, washed with saturated NaCl aqueous solution, dried over Na_2_SO_4_, and concentrated. The residue was purified by silica gel column chromatography (ethyl acetate) to give the desired product Biotin-ZHSI-1-4 (80 mg, 66%), which is a yellow solid. ^1^H NMR spectrum: (400 MHz, DMSO-*d6*) δ 9.40 (s, 1 H), 8.67 (s, 1 H), 8.65 (s, 1 H), 8.06–7.96 (m, 1H), 7.88 (d, *J* = 9.6 Hz, 1 H), 7.85–7.78 (m, 1 H), 7.20 (s, 1 H), 6.68–6.60 (m, 2 H), 5.94 (s, 1 H), 5.87–5.78 (m, 1 H), 4.17–4.07 (m, 1 H), 3.85 (s, 2 H), 2.14 (s, 3 H), 1.38 (s, 9 H), 1.21 (d,*J* = 6.4 Hz, 6 H).

#### Biotin-ZHSI-1-5: Tert-butyl(3-(6-((2-(isopropylamino)-6-methylpyrimidin-4-yl) amino) quinolin-3-yl) propyl) carbamate

First, 10% Pd/C (8 mg) was added to a solution of Biotin-318-4 (80 mg, 0.18 mmol) in CH_3_OH (2 ml). The mixture was stirred under an H_2_ atmosphere overnight. The solvent was filtered, and the filtrate was concentrated to give the desired product, Biotin-318-5 (80 mg, 100%), which is a yellow solid. ^1^H NMR spectrum: (400 MHz, CDCl_3_) δ 8.70 (s, 1 H), 8.04 (d, *J* = 9.2 Hz, 1 H), 7.98 (s, 1 H), 7.83 (s, 1 H), 7.59 (d,*J* = 8.8 Hz, 1 H), 7.05–6.98 (m, 1 H), 5.89 (s, 1 H), 4.65–4.57 (m, 1 H), 4.20–4.10 (m, 1 H), 3.38 (t, *J* = 6.8 Hz, 1 H), 3.27–3.17 (m, 2 H), 2.09 (s, 3 H), 2.05–2.00 (m, 1 H), 1.95–1.90 (m, 1 H), 1.45 (s, 9 H), 1.30 (d, *J* = 6.8 Hz, 6 H).

#### Biotin-ZHSI-1-6: *N*^*4*^-(3-(3-Aminopropyl) quinolin-6-yl)-*N*^*2*^-isopropyl-6-methylpyrimidine-2,4-diamine hydrochloride (Biotin-318-3)

3-M HCl methanol solution (1 ml, 3 mmol) was added to a mixture of Biotin-318-5 (80 mg, 0.18 mmol) in CH_3_OH (1 ml). The mixture was stirred at room temperature for 5 h. The solvent was concentrated to give the desired product, Biotin-318-3 (70 mg, 100%), which is a yellow solid.

#### Biotin-ZHSI-1: *N*-(3-(6-((2-(isopropylamino)-6-methylpyrimidin-4-yl) amino) quinolin-3-yl) propyl)-6-(5-((3aS,4S,6aR)-2-oxohexahydro-*1H*-thieno[3,4-*d*] imidazol-4-yl) pentanamido) hexanamide

Biotin-NC-3 (123 mg, 0.27 mmol) and Et_3_N (50 mg, 0.5 mmol) were added to a solution of Biotin-ZHSI-1-6 (70 mg, 0.18 mmol) in DMF (1 ml). The mixture was stirred at room temperature overnight. The solvent was removed under vacuum and the residue was purified by silica gel column chromatography (dichloromethane/methanol/NH_4_OH = 100/10/1) to give the desired product, Biotin-ZHSI-1 (28 mg, 22%), which is a yellow solid. ^1^H NMR spectrum: (400 MHz, DMSO-*d*_*6*_) δ 9.44 (br s, 1 H), 8.59 (s, 1 H), 8.56 (br s, 1 H), 7.94 (br s, 1 H), 7.86 (d, *J* = 8.8 Hz, 1 H), 7.85 (s, 1 H), 7.78 (d, *J* = 8.8 Hz, 1 H), 7.75–7.70 (m, 1 H), 8.68 (br s, 1 H), 6.42 (s, 1 H), 6.36 (s, 1 H), 5.96 (s, 1 H),4.32–4.26 (m, 1 H), 4.16–4.06 (m, 2 H), 3.15–3.05 (m, 3 H), 3.04–2.97 (m, 2H), 2.84–2.73 (m, 3 H), 2.57 (d, *J* = 12.4 Hz, 1 H), 2.15 (s, 3 H), 2.10–1.98 (m, 4 H), 1.85–1.75 (m, 2 H), 1.65–1.55 (m, 1H), 1.53–1.43 (m, 4 H), 1.42–1.33 (m, 2 H), 1.30–1.18 (m, 10 H).

### Statistical analysis

The GraphPad Prism software was used for all statistical analyses. An unpaired two-tailed Student’s *t* test was performed for two-group comparisons. Statistical significance was accepted for P values <0.05 and the results are represented as means ± SEM.

### Online supplemental material

Fig. S1 contains the process of CRISPR-Cas9 screening and the inhibitory effect of knocking down LAMTOR2 and LAMTOR4 on EV71-induced cell death. Fig. S2 includes qPCR and DNA sequence alignment to demonstrate that the Ragulator-Rag complex is required for EV71 replication. Fig. S3 shows that 3D directly interacts with RagB rather than with other components of the Ragulator-Rag complex. Fig. S4 shows that deletion of LAMTOR3 does not affect the viral titers of HSV and VSV. Fig. S5 shows the synthesis steps of ZHSI-1 and Biotin-ZHSI-1, as well as their inhibition of viral protein expression of EV71 and CVA16. Table S1 shows the primer sequences for qPCR.

## Supplementary Material

Table S1 lists primer sequences for qPCR.

SourceData F1 is the source file for Fig. 1.

SourceData F2 is the source file for Fig. 2.

SourceData F3 is the source file for Fig. 3.

SourceData F4 is the source file for Fig. 4.

SourceData F5 is the source file for Fig. 5.

SourceData F6 is the source file for Fig. 6.

SourceData FS1 is the source file for Fig. S1.

SourceData FS2 is the source file for Fig. S2.

SourceData FS3 is the source file for Fig. S3.

SourceData FS5 is the source file for Fig. S5.
