# Supplementary figures and images for: Inhibition of lysosome-tethered Ragulator-Rag-3D complex restricts the replication of Enterovirus 71 and Coxsackie A16
Source: J Cell Biol. 2023 Oct 31;222(12):e202303108. doi: 10.1083/jcb.202303108 (PMC10619577; doi:10.1083/jcb.202303108)

**B**

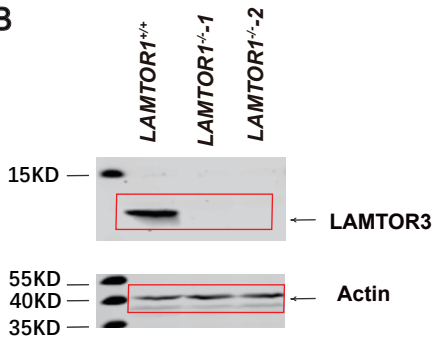

**C**

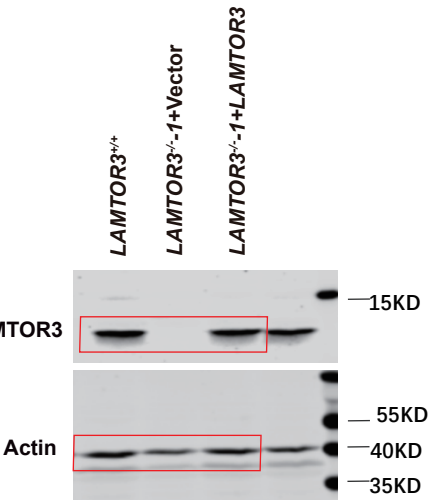

**E**

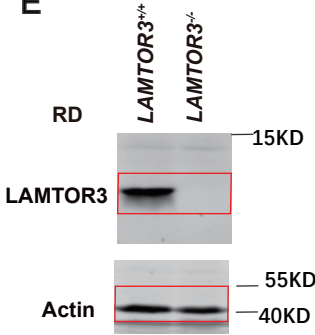

**G**

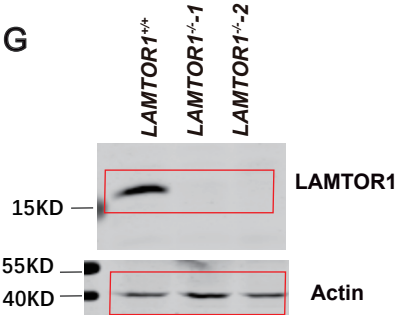

**J**

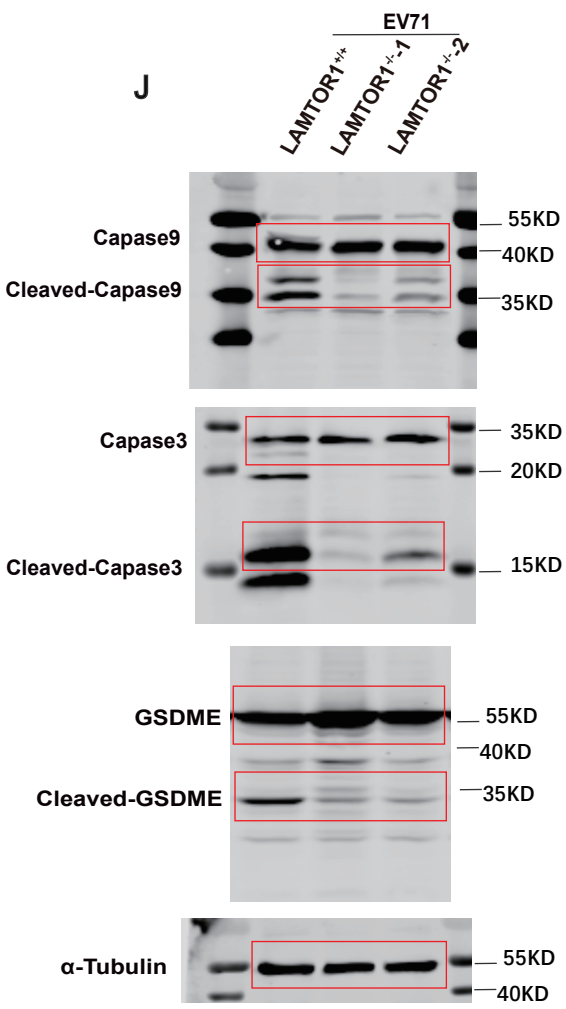

**I**

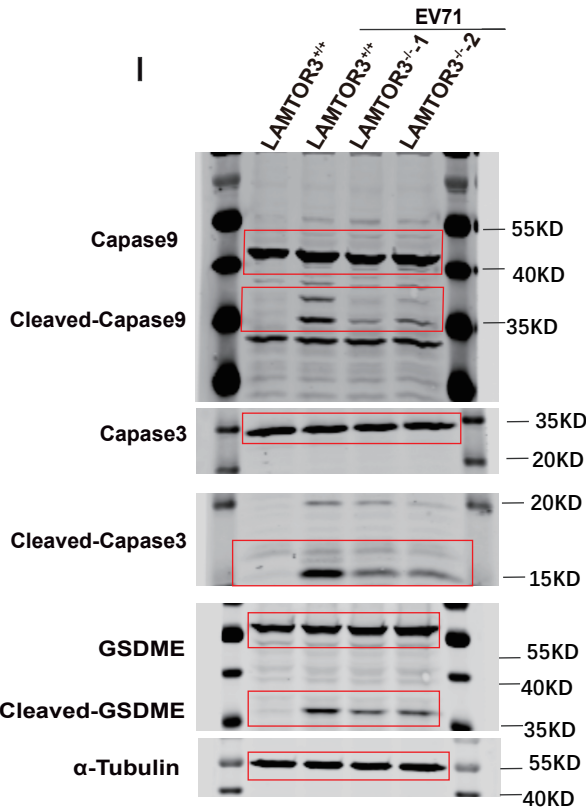

Supplement: SourceData F1 — is the source file for Fig. 1. [file JCB_202303108_SourceDataF1.pdf]

SourceData F2

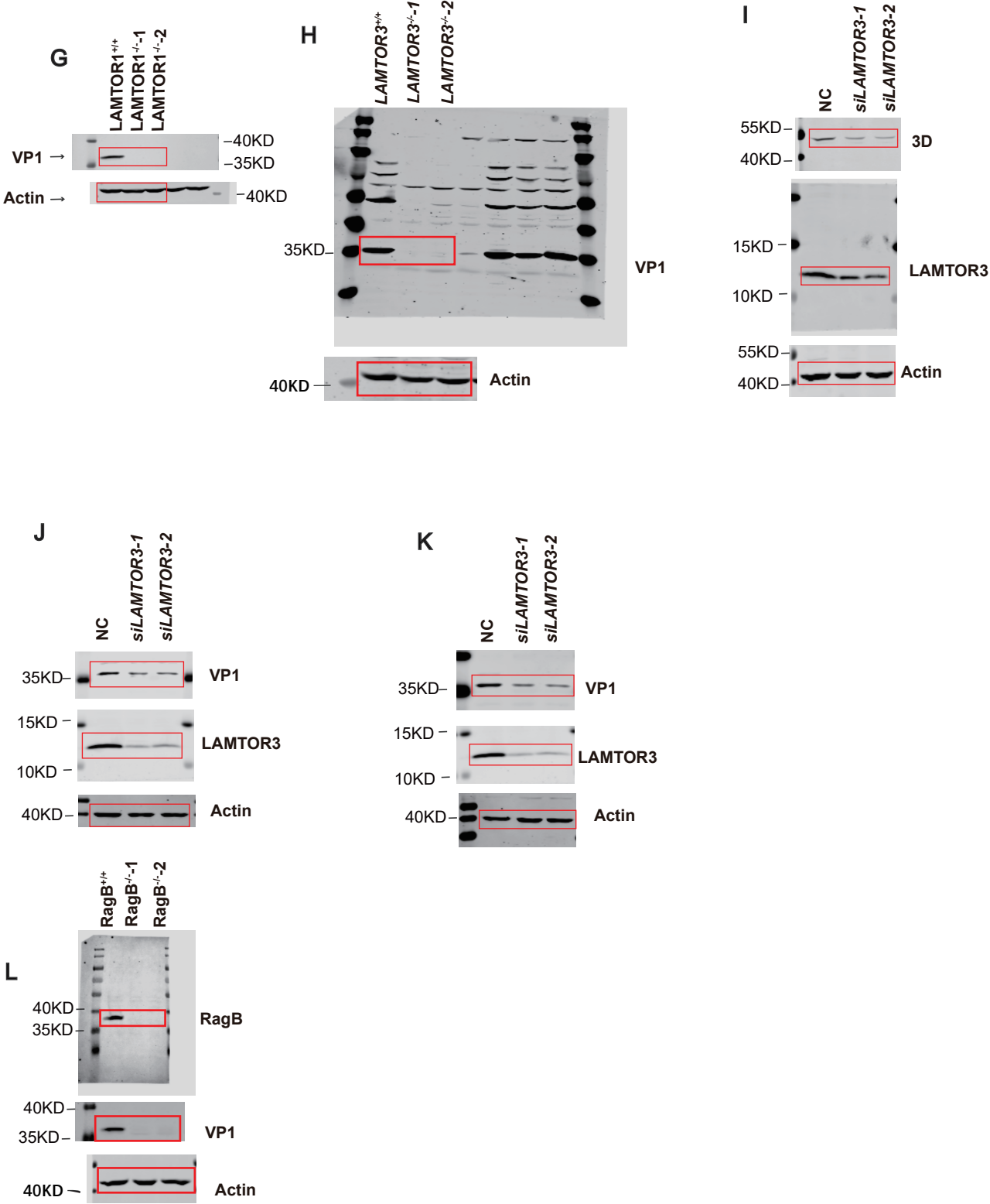

Supplement: SourceData F2 — is the source file for Fig. 2. [file JCB_202303108_SourceDataF2.pdf]

**B**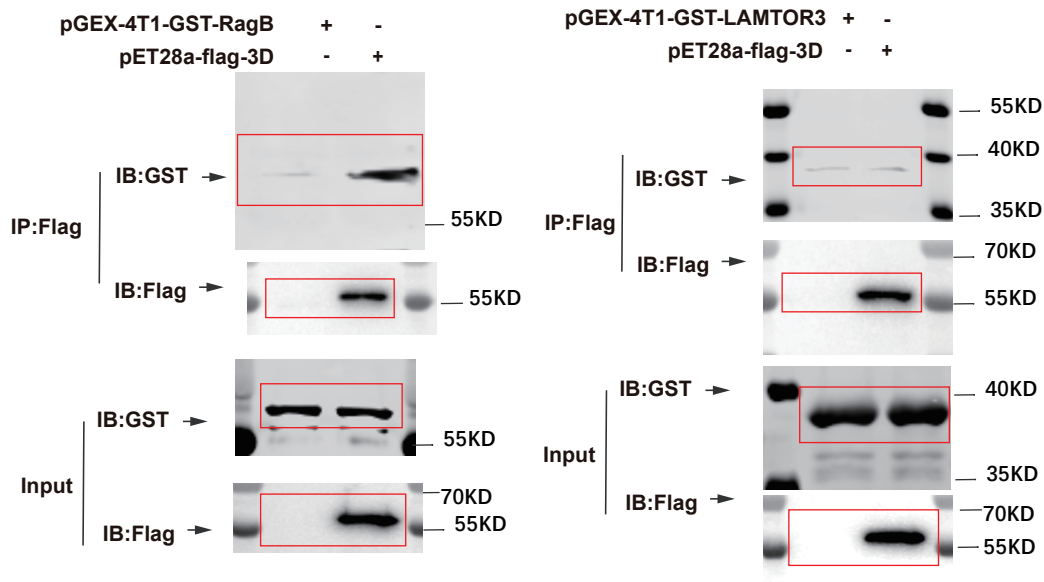**C**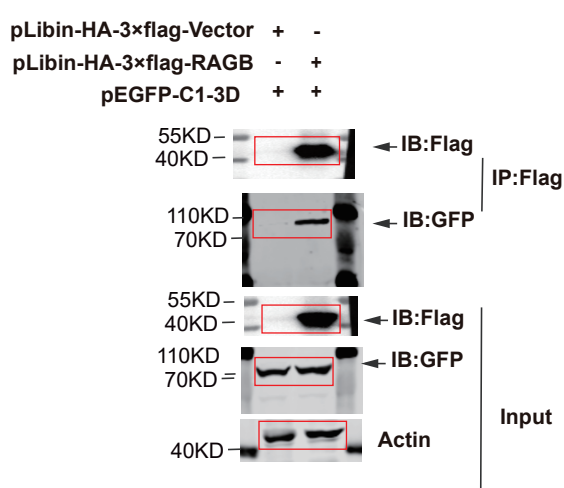**F**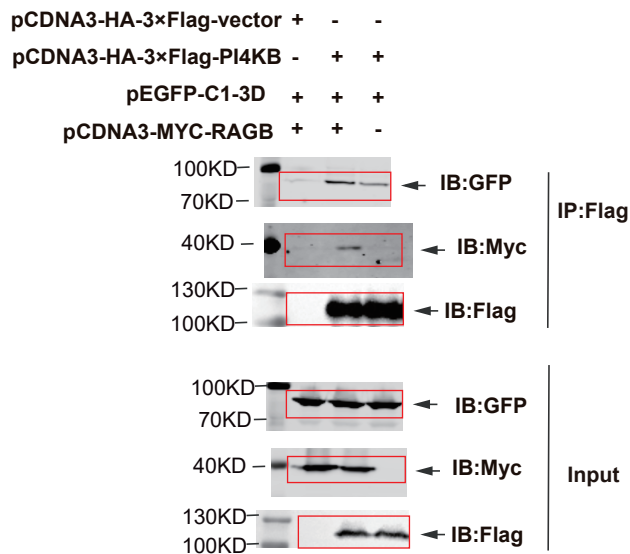

Supplement: SourceData F3 — is the source file for Fig. 3. [file JCB_202303108_SourceDataF3.pdf]

**B**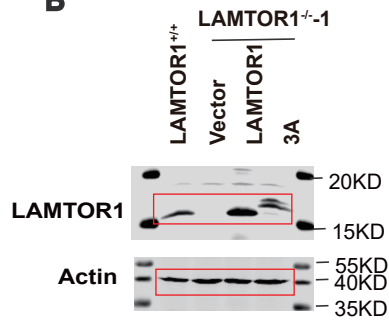

Supplement: SourceData F4 — is the source file for Fig. 4. [file JCB_202303108_SourceDataF4.pdf]

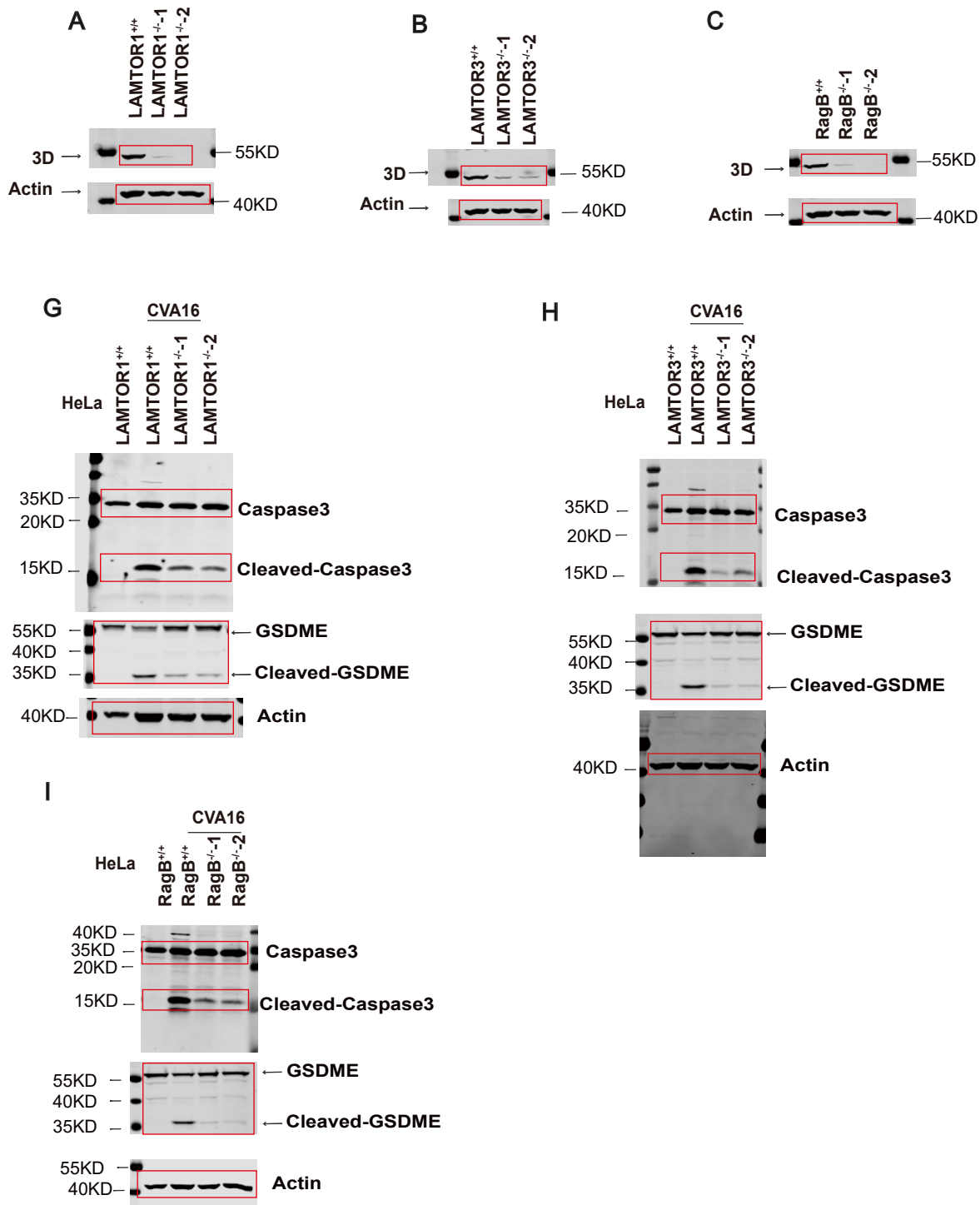

Supplement: SourceData F5 — is the source file for Fig. 5. [file JCB_202303108_SourceDataF5.pdf]

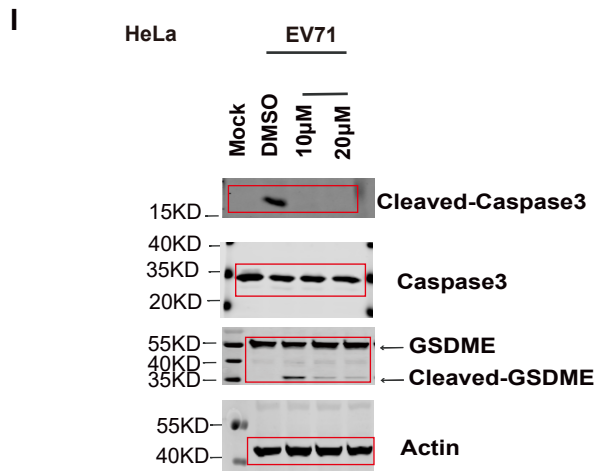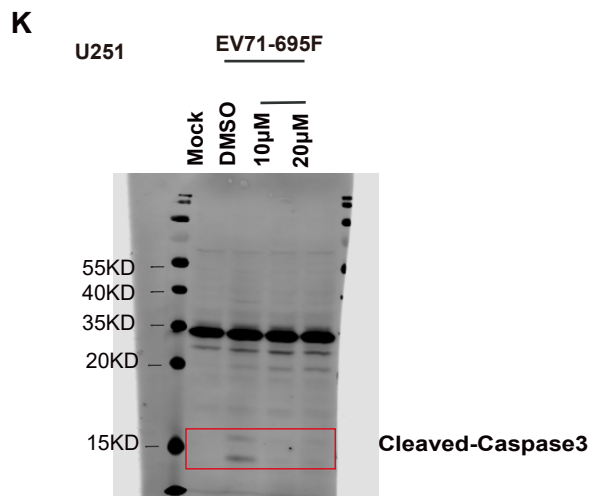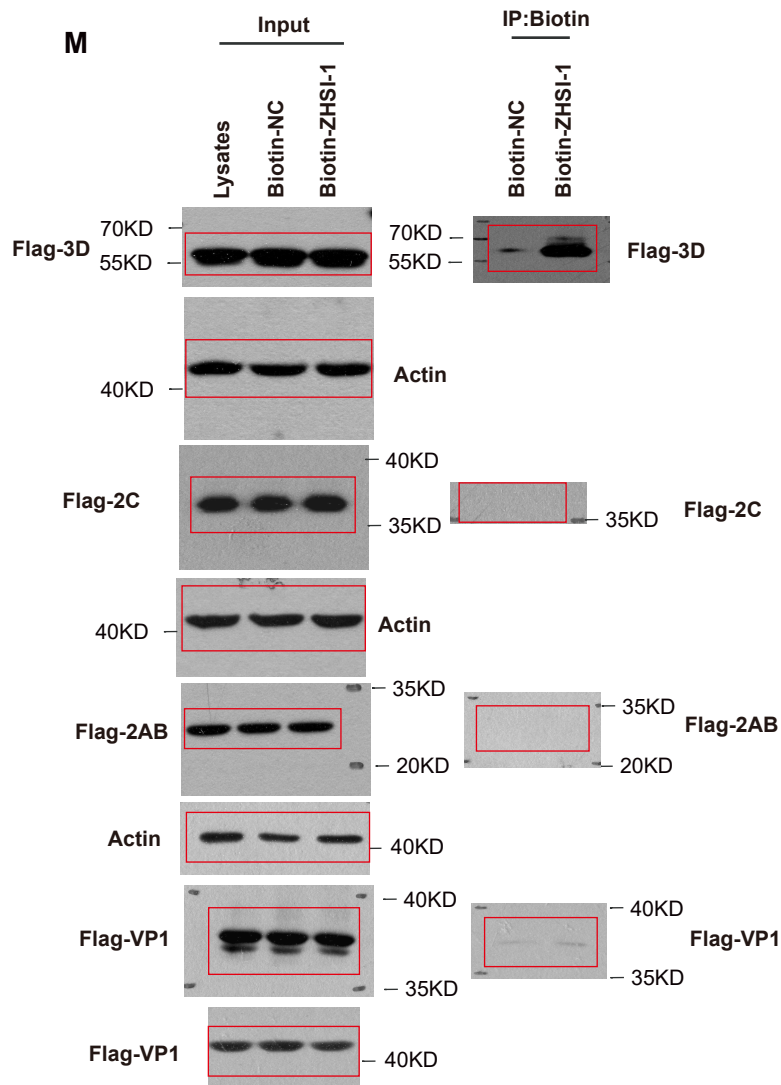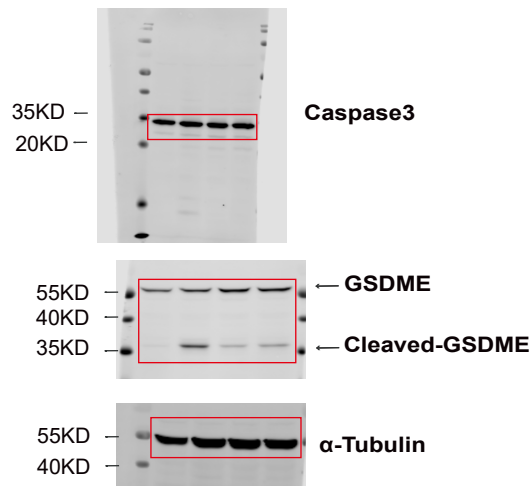

Supplement: SourceData F6 — is the source file for Fig. 6. [file JCB_202303108_SourceDataF6.pdf]

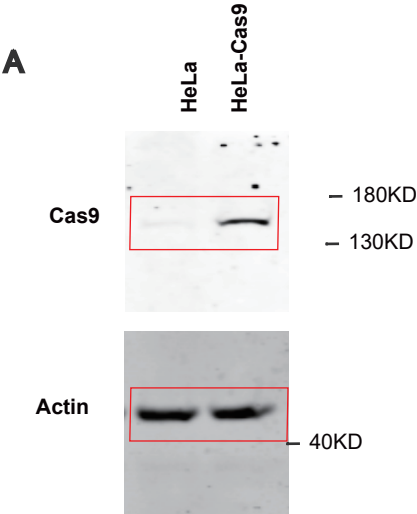

Supplement: SourceData FS1 — is the source file for Fig. S1. [file JCB_202303108_SourceDataFS1.pdf]

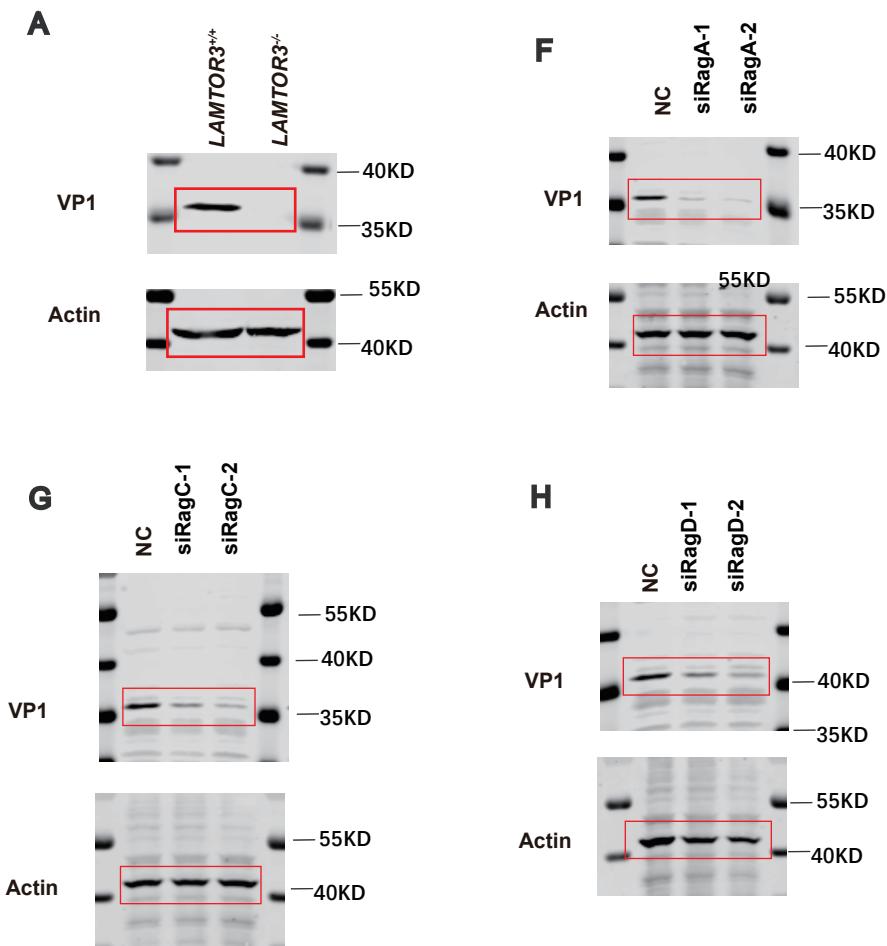

Supplement: SourceData FS2 — is the source file for Fig. S2. [file JCB_202303108_SourceDataFS2.pdf]

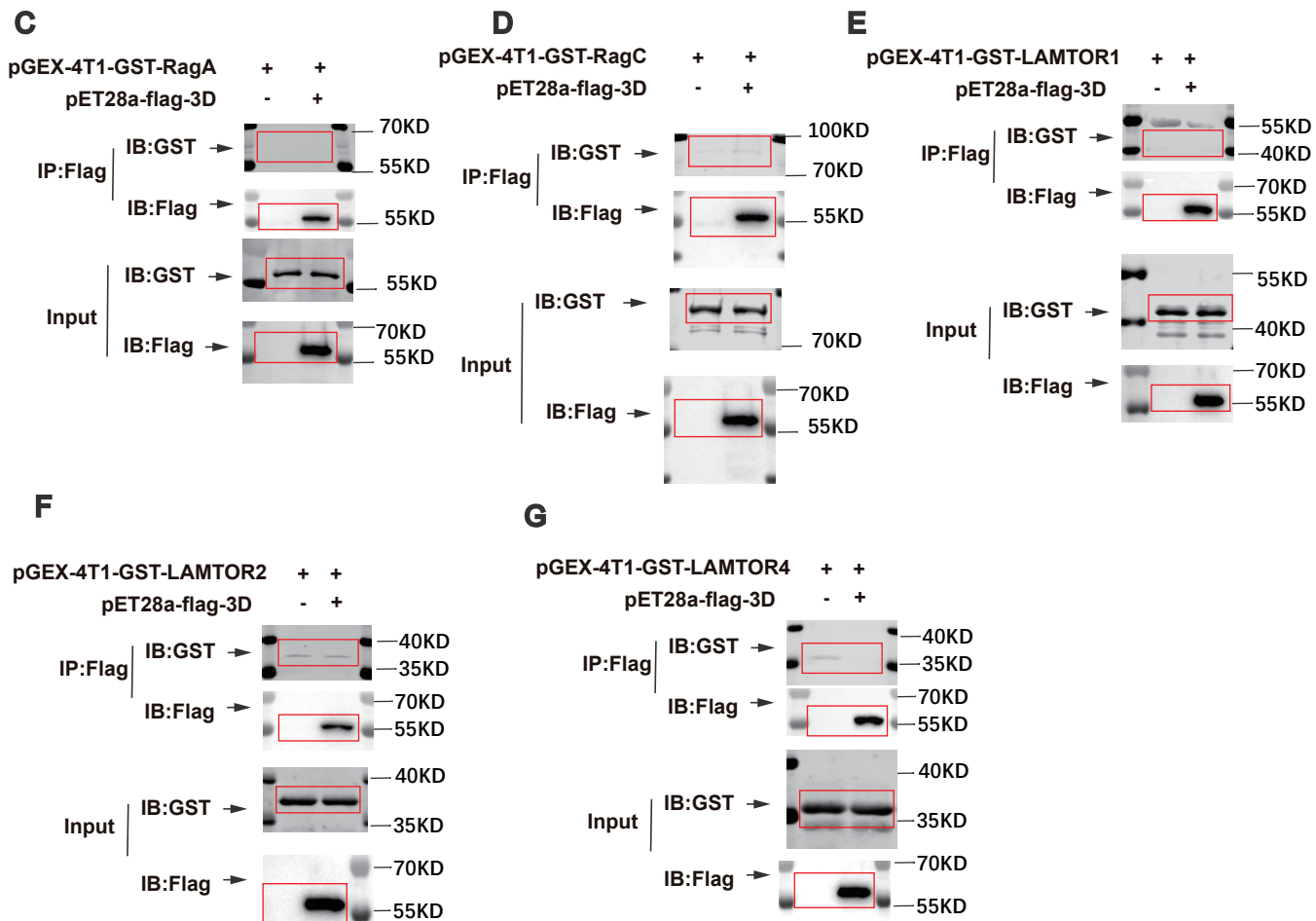

Supplement: SourceData FS3 — is the source file for Fig. S3. [file JCB_202303108_SourceDataFS3.pdf]

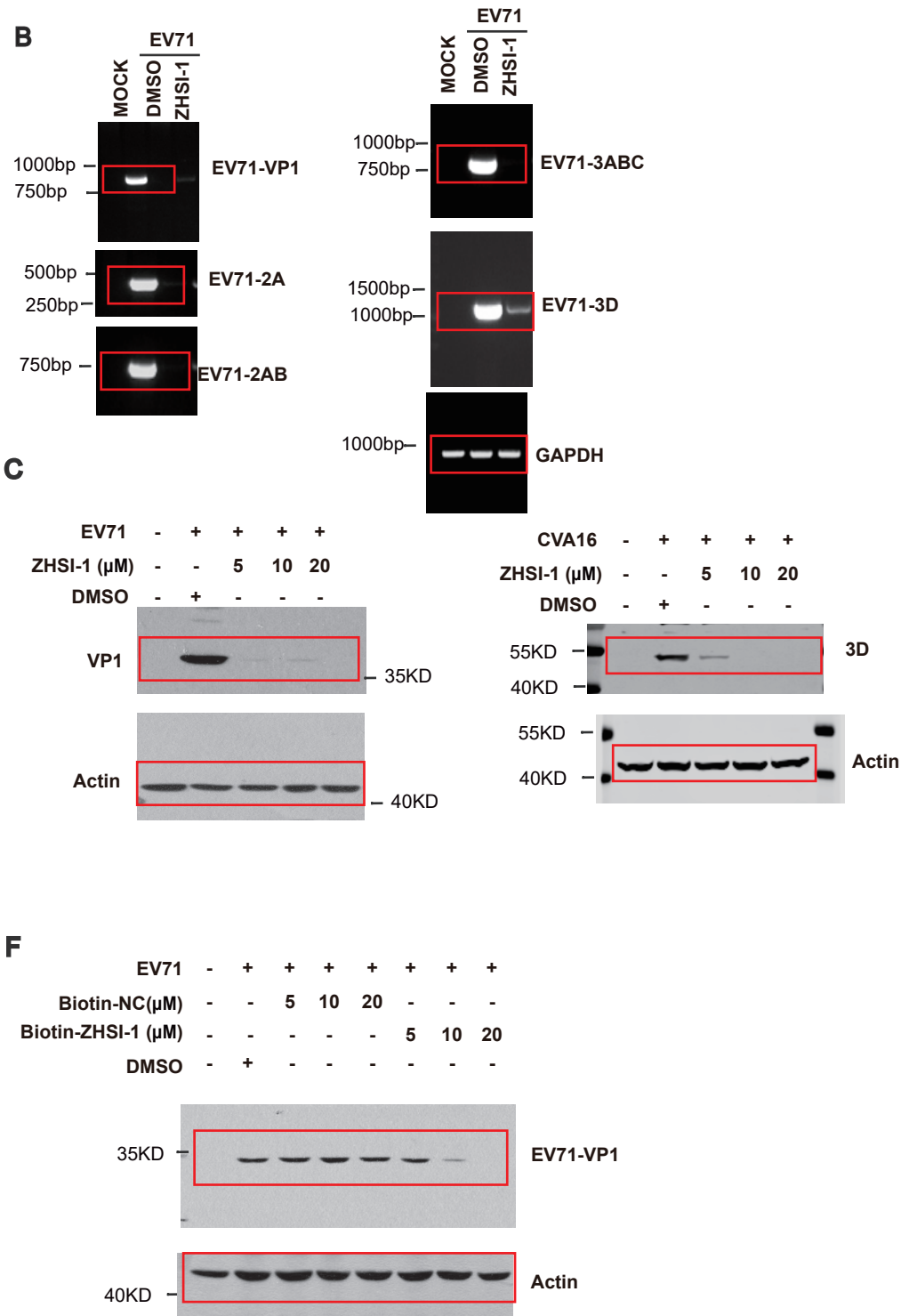

Supplement: SourceData FS5 — is the source file for Fig. S5. [file JCB_202303108_SourceDataFS5.pdf]
